# Yerba mate (*Ilex paraguariensis*) genome provides new insights into convergent evolution of caffeine biosynthesis

**DOI:** 10.7554/eLife.104759

**Published:** 2025-01-08

**Authors:** Federico A Vignale, Andrea Hernandez Garcia, Carlos P Modenutti, Ezequiel J Sosa, Lucas A Defelipe, Renato Oliveira, Gisele L Nunes, Raúl M Acevedo, German F Burguener, Sebastian M Rossi, Pedro D Zapata, Dardo A Marti, Pedro Sansberro, Guilherme Oliveira, Emily M Catania, Madeline N Smith, Nicole M Dubs, Satish Nair, Todd J Barkman, Adrian G Turjanski

**Affiliations:** 1 European Molecular Biology Laboratory - Hamburg Unit Hamburg Germany; 2 Department of Biochemistry, University of Illinois at Urbana-Champaign Urbana United States; 3 IQUIBICEN-CONICET, Ciudad Universitaria, Pabellón 2 Ciudad Autonoma de Buenos Aires Argentina; 4 Departamento de Química Biológica, Facultad de Ciencias Exactas y Naturales, Universidad de Buenos Aires, Ciudad Universitaria, Pabellón 2 Ciudad Autónoma de Buenos Aires Argentina; 5 Instituto Tecnológico Vale Belém Brazil; 6 Laboratorio de Biotecnología Aplicada y Genómica Funcional, Instituto de Botánica del Nordeste (IBONE-CONICET), Facultad de Ciencias Agrarias, Universidad Nacional del Nordeste Corrientes Argentina; 7 Department of Plant Sciences, University of California, Davis Davis United States; 8 Instituto de Biotecnología de Misiones, Facultad de Ciencias Exactas, Químicas y Naturales, Universidad Nacional de Misiones (INBIOMIS-FCEQyN-UNaM) Misiones Argentina; 9 Instituto de Biología Subtropical, Universidad Nacional de Misiones (IBS-UNaM-CONICET) Posadas Argentina; 10 Department of Biological Sciences, Western Michigan University Kalamazoo United States; 11 Carl R. Woese Institute for Genomic Biology, University of Illinois at Urbana-Champaign Urbana United States; 12 Center for Biophysics and Quantitative Biology, University of Illinois at Urbana Champaign Urbana United States; Max Planck Institute for Biology Tübingen Germany; Max Planck Institute for Biology Tübingen Germany

**Keywords:** *Ilex paraguariensis*, yerba mate, genome, caffeine, convergent evolution, whole-genome duplication, Other

## Abstract

Yerba mate (YM, *Ilex paraguariensis*) is an economically important crop marketed for the elaboration of mate, the third-most widely consumed caffeine-containing infusion worldwide. Here, we report the first genome assembly of this species, which has a total length of 1.06 Gb and contains 53,390 protein-coding genes. Comparative analyses revealed that the large YM genome size is partly due to a whole-genome duplication (Ip-α) during the early evolutionary history of *Ilex*, in addition to the hexaploidization event (γ) shared by core eudicots. Characterization of the genome allowed us to clone the genes encoding methyltransferase enzymes that catalyse multiple reactions required for caffeine production. To our surprise, this species has converged upon a different biochemical pathway compared to that of coffee and tea. In order to gain insight into the structural basis for the convergent enzyme activities, we obtained a crystal structure for the terminal enzyme in the pathway that forms caffeine. The structure reveals that convergent solutions have evolved for substrate positioning because different amino acid residues facilitate a different substrate orientation such that efficient methylation occurs in the independently evolved enzymes in YM and coffee. While our results show phylogenomic constraint limits the genes coopted for convergence of caffeine biosynthesis, the X-ray diffraction data suggest structural constraints are minimal for the convergent evolution of individual reactions.

## Introduction

In the genomic era, hundreds of plant species have had their nucleotide sequences determined to provide unprecedented insight into the genetic basis of many traits. One of the few species of economic importance for which no genomic data exist is *Ilex paraguariensis* var. *paraguariensis* A. St. Hilaire (Aquifoliaceae), colloquially known as yerba mate (YM), which is a caffeinated diploid tree-species (2*n* = 2*x* = 40) endemic to the subtropical rainforests of South America (Niklas, 1987). The dried leaves and twigs of this dioecious evergreen are used to prepare a traditional infusion named mate, or chimarrão, widely consumed around the world. Approximately 300,000 ha are cultivated with this tree crop, with Argentina responsible for 80% of worldwide production (Heck and de Mejia, 2007). The mate infusion has been shown to have numerous beneficial effects in humans including as an antioxidant (Sánchez Boado et al., 2015; Gugliucci, 1996; Vieira et al., 2010), antidiabetic (Kang et al., 2012; Ríos et al., 2015), as well as central nervous system stimulant (Santos et al., 2015), among others. Several bioactive compounds have been identified in YM that might be responsible for its effects, including terpenes, flavonoids, phenolics, and methylxanthines (Heck and de Mejia, 2007). Although its stimulant properties are best known and mostly related to caffeine content, little is known about the genetic and biochemical mechanisms of how YM synthesizes this, or any, of its important metabolites. Despite the recent release of three other *Ilex* genome sequences (Kong et al., 2022; Xu et al., 2022; Yao et al., 2022), none of the species produce caffeine, making the genetic basis for convergent evolution of this trait in YM unclear.

**Figure 1.**
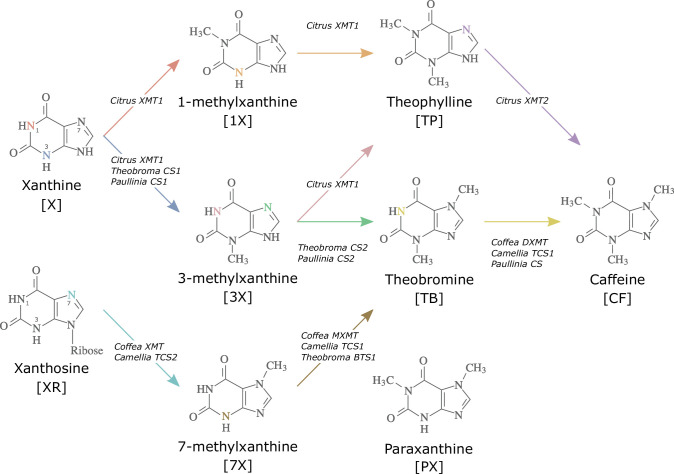
Biosynthetic routes to caffeine within the xanthine alkaloid network. CF, caffeine; PX, paraxanthine; TB, theobromine; TP, theophylline; 1X, 1-methylxanthine; 3X, 3-methylxanthine; 7X, 7-methylxanthine; XR, xanthosine; X, xanthine. Nitrogen atoms are coloured to match the arrows corresponding to the enzymes that methylate them. Adapted with permission from O’Donnell et al., 2021.

Convergent evolution has occurred throughout the tree of life and is particularly rampant in plants (Sackton and Clark, 2019) where examples of repeated origins of morphological (Thorogood et al., 2018), anatomical (Wan et al., 2018), physiological (Yang et al., 2017), and biochemical (Pichersky and Lewinsohn, 2011) traits have been documented. Caffeine (CF) is a xanthine alkaloid that has independently evolved no less than six times across angiosperms and has implications for pollination, insect defence, and allelopathy (Anaya et al., 2006; Stevenson et al., 2017). There are multiple biosynthetic routes to caffeine possible within the xanthine alkaloid network (Figure 1). Within the Rosid genera *Theobroma*, *Paullinia*, and *Citrus*, sequential methylation of xanthine (X), 1- and/or 3-methylxanthine (1X, 3X) and, finally, either theophylline (TP) or theobromine (TB) leads to caffeine (Huang et al., 2016). In contrast, the Asterids, *Coffea* and *Camellia*, appear to sequentially methylate xanthosine (XR), 7-methylxanthine (7X), and theobromine (TB) to yield caffeine (Ashihara et al., 1996; Suzuki and Takahashi, 1976; Figure 1). Regardless of which pathway is utilized, species differ in terms of which SABATH enzyme family members were convergently recruited to synthesize caffeine: xanthine methyltransferase (XMT) is used by *Citrus* and *Coffea* while the paralogous caffeine synthase (CS) is used by *Camellia*, *Theobroma*, and *Paullinia* (Huang et al., 2016; Kato et al., 1996; Uefuji et al., 2003; Figure 1). Convergence appears to also extend to the mutational level, since different amino acid replacements to homologous regions of CS and XMT enzymes appear to govern the evolution of substrate preference switches (O’Donnell et al., 2021). However, it remains unclear whether mutations lead to convergent three-dimensional protein structures to confer convergent substrate interactions and catalysis by the enzymes. Because XMT- and CS-type enzymes have been convergently recruited in both Rosids and Asterids to catalyse the same or different pathways, it suggests considerable evolutionary lability underlying caffeine production in plants (Huang et al., 2016). As a result, it is difficult to predict what sets of genes, biochemical reactions, and structural properties might lead to the evolution of caffeine biosynthesis in YM, or any plant, a priori.

Although some transcriptomic resources have been generated for YM (Acevedo et al., 2019; Debat et al., 2014; Fay et al., 2018), a complete genome sequence has the potential to advance our understanding of the metabolic potential of this important crop and facilitate improvement. Here, we describe the first draft genome of YM and report on its composition, organization, and evolution. The genomic sequence enabled us to uncover the genetic, biochemical, mutational, and structural bases for convergent evolution of caffeine in YM. Our comparative analyses of caffeine-producing enzymes across angiosperms reveal how convergence may be the result of constrained evolutionary genomic potential but relatively unconstrained structural potential.

## Results and discussion

### YM genome sequencing, assembly, and annotation

The YM genome was sequenced combining Illumina and PacBio sequencing technologies. With Illumina sequencing, we generated ~263.2 Gb of short reads from various DNA fragment sizes (350 bp, 550 bp, 3 kbp, 8 kbp, and 12 kbp), while with PacBio sequencing, we generated ~77.5 Gb of long reads. These reads represent ~158.5- and ~49.3-fold base-pair coverage of the genome, respectively (Table 1). The total assembly length was ~1.06 Gb and consisted of 10,611 scaffolds (≥1 kb) with an N50 length of ~510.8 kb (Table 2). To assess the completeness of the genome, we aligned the available YM transcriptome reads (Acevedo et al., 2019; Debat et al., 2014; Fay et al., 2018) and the YM genomic short reads generated in this study with the assembly: 99.3% of the former and 99.5% of the latter were mapped. The GC content of the genome assembly was 36.33% (Table 2), similar to that of other eudicots (33.70–38.20 GC%) (Singh et al., 2016) and almost identical to that of *Ilex polyneura* (36.08 GC%) (Yao et al., 2022), *Ilex asprella* (36.25 GC%) (Kong et al., 2022), and *Ilex latifolia* (36.44 GC%) (Xu et al., 2022), the only three *Ilex* species with sequenced genomes. About 64.63% of the genome assembly was composed of repetitive sequences, of which ~36.22% were retrotransposons, ~1.80% were DNA transposons, ~0.74% were simple repeats, and ~0.15% were low complexity regions. Long terminal-repeat retrotransposons of the Gypsy and Copia families were the most abundant transposable elements, as observed in many sequenced plant genomes (Galindo-González et al., 2017), followed by long interspersed nuclear elements (LINEs) and hobo-Activator transposons, among others (Table 3).

**Table 1.** Statistics of the genome sequencing data of yerba mate.

Library	Number of reads	Read length	Total length	Coverage
Pair-end 350 bp #1	360,653,408	101	36.4 Gbp	21.8×
Pair-end 350 bp #2	368,746,464	101	37.2 Gbp	22.3×
Pair-end 550 bp	356,261,246	101	36 Gbp	21.5×
Mate-pair 3 kbp #1	415,398,586	101	30.3 Gbp	18.2×
Mate-pair 3 kbp #2	410,588,934	101	30 Gbp	17.9×
Mate-pair 3 kbp #3	343,059,350	101	25 Gbp	15×
Mate-pair 8 kbp	393,202,256	101	34.6 Gbp	20.7×
Mate-pair 12 kbp	415,478,776	101	33.7 Gbp	20.1×
PacBio long reads	19,514,627	50 bp to 61 kbp	77.5 Gbp	49.3×
Total			341 Gbp	207.8×

**Table 2.** Statistics of the genome assembly of yerba mate.

Metric	Value
# scaffolds (≥1000 bp)	10,611
# scaffolds (≥5000 bp)	9343
# scaffolds (≥10,000 bp)	8951
# scaffolds (≥25,000 bp)	5944
# scaffolds (≥50,000 bp)	2595
Total length (≥50,000 bp)	887,124,725
# scaffolds	10,611
Largest scaffold	7,402,063
Total length	1,064,802,823
GC (%)	36.33
N50	510,878
N75	132,523
L50	506
L75	1461
# N’s per 100 kbp	1976.99

**Table 3.** Classification and distribution of repetitive DNA elements in yerba mate.

	Number	Length occupied (bp)	Percentage of the genome (%)
Class I retrotransposons	421,599	385,714,532	36.22
SINEs	840	154,298	0.01
Penelope	0	0	0.00
LINEs	35,433	17,109,207	1.61
CRE/SLACS	0	0	0.00
L2/CR1/Rex	575	135,549	0.01
R1/LOA/Jockey	443	76,937	0.01
R2/R4/NeSL	0	0	0.00
RTE/Bov-B	8599	2,126,765	0.20
L1/CIN4	25,816	14,769,956	1.39
LTR retrotransposons	385,326	368,451,027	34.60
BEL/Pao	709	266,632	0.03
Ty1/Copia	98,237	67,631,136	6.35
Gypsy/DIRS1	216,472	274,526,515	25.78
Retroviral	0	0	0.00
Class II DNA transposons	45,427	19,116,209	1.80
hobo-Activator	21,335	6,378,850	0.60
Tc1-IS630-Pogo	0	0	0.00
En-Spm	0	0	0.00
MuDR-IS905	0	0	0.00
PiggyBac	0	0	0.00
Tourist/Harbinger	5870	2,846,548	0.27
Others	0	0	0.00
Unclassified	990,080	269,430,122	25.30
Total interspersed repeats	674,260	863	63.32
Small RNA	4362	718,762	0.07
Satellites	0	0	0.00
Simple repeats	185,507	7,911,080	0.74
Low complexity	31,856	1,606,255	0.15

A total of 53,390 protein-coding genes were predicted in the genome, with a mean coding sequence length of 3062 bp and 4.23 exons per gene. Of these, 41,483 (~77.63%) could be annotated with GO terms, EC numbers or Pfam domains. In addition, we identified 4530 non-coding RNA genes, including 2670 small nucleolar RNAs, 815 transfer RNAs, 471 ribosomal RNAs, 348 small nuclear RNAs, and 226 micro RNAs (Appendix 1, Appendix 1—tables 1–3). To further assess the completeness of the assembly, we aligned the scaffolds with the KOG (Tatusov et al., 2003) and DEG (Luo et al., 2014) databases, determining that 98% of the core gene families from the KOG database and 97.5% of the *Arabidopsis thaliana* DEG subset were present. Then, we performed a Benchmarking Universal Single-Copy Orthologs (BUSCO) (Manni et al., 2021) assessment using the eudicot ODB10 database. Among 2326 conserved single-copy genes, ~96.20% were retrieved, of which ~78.80% were complete and single copies, ~17.40% were complete and in duplicates, ~3.10% were fragmented, and only ~0.70% were missing. These results suggest that the coding region of the assembly is nearly complete. The number of estimated genes for YM is higher than the ca. 39,000 reported from the genome sequences of other *Ilex* species (Kong et al., 2022; Xu et al., 2022; Yao et al., 2022). This could be at least partly due to the larger genome size of YM as estimated from flow cytometry relative to the other species (Gottlieb and Poggio, 2015).

### Evolutionary analysis of YM genome provides evidence of whole-genome duplication in an early *Ilex* ancestor

**Figure 2.**
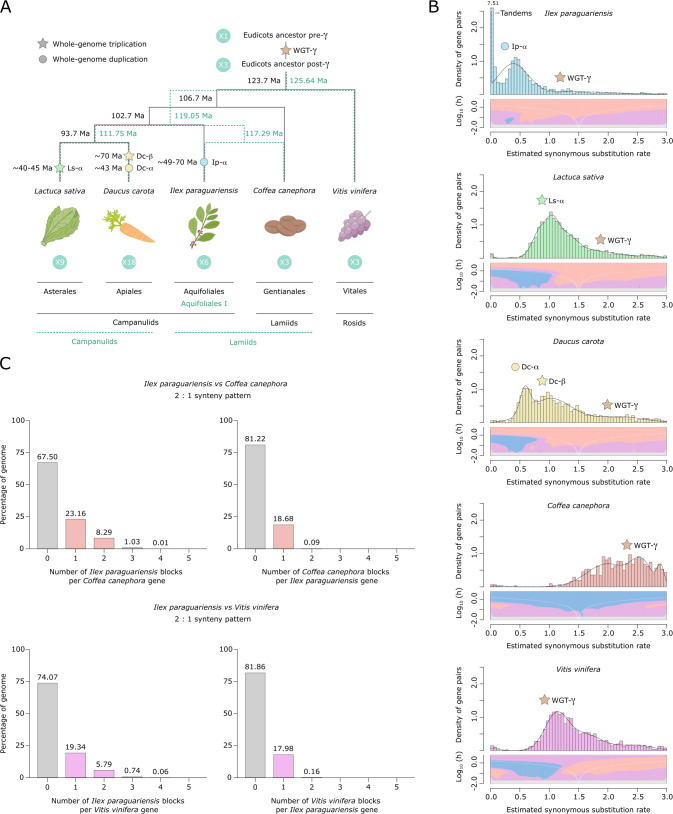
Yerba mate genome duplication history. (**A**) Evolutionary scenario of the eudicot genomes of *Lactuca sativa*, *Daucus carota*, *Ilex paraguariensis*, *Coffea canephora*, and *Vitis vinifera*, from their ancestor pre-γ. The plastid genome phylogeny is represented with solid black lines, while the multiple nuclear genome phylogeny is represented with green dashed lines. Paleopolyploidizations are shown with coloured dots (duplications) and stars (triplications). Divergence time estimates for the lineages, as well as age estimates for the *L. sativa* and *D. carota* paleopolyploidizations were obtained from the literature (Iorizzo et al., 2016; Magallón et al., 2015; Reyes-Chin-Wo et al., 2017; Zhang et al., 2020b). Ma, million years ago. (**B**) *K*_s_ distributions with Gaussian mixture model and SiZer analyses of *I. paraguariensis* (blue), *L. sativa* (green), *D. carota* (yellow), *C. canephora* (red), and *V. vinifera* (purple) paralogues. SiZer maps below histograms identify significant peaks at corresponding *K*_s_ values. Blue represents significant increases in slope, red indicates significant decreases, purple represents no significant slope change, and grey indicates not enough data for the test. (**C**) Comparative genomic synteny analyses of *I. paraguariensis* with *C. canephora* and *V. vinifera*.

Most plant lineages have experienced ancient polyploidization events followed by massive duplicate gene losses and genome rearrangements, which may have contributed to the evolution of developmental and metabolic complexity (Landis et al., 2018; Sankoff and Zheng, 2018). Recent transcriptome-based analyses (One Thousand Plant Transcriptomes Initiative, 2019; Zhang et al., 2020b) reported an ancient polyploidization event in the *Ilex* lineage around 60 Ma (Cretaceous–Paleogene boundary), based on phylogenomic and synonymous substitution rate (*K*_s_) evidence. Evolutionary analyses of *I. polyneura* (Yao et al., 2022) and *I. latifolia* (Xu et al., 2022) genomes also provided evidence of a shared *Ilex*-specific whole-genome duplication (WGD). As YM is the first American holly to have its genome sequenced, we performed synteny-based analyses of its genome to deepen our understanding of Aquifoliales evolution (Figure 2, Figure 2—figure supplement 1). The *K*_s_ distribution of YM paralogues (Figure 2B) revealed a significant peak with a median *K*_s_ value of ~0.37, not shared with the rest of the eudicot genomes analysed (Figure 2B, Figure 2—figure supplement 1). This confirms the lineage-specific polyploidization event (Ip-α) previously reported in *Ilex* (One Thousand Plant Transcriptomes Initiative, 2019; Xu et al., 2022; Yao et al., 2022; Zhang et al., 2020b), in addition to the shared ancestral WGT-γ which is indicated by a median *K*_s_ value of ~1.4 (Figure 2B). A WGD in the common ancestor of *Ilex* species is further supported by 2:1 syntenic depth ratios between the YM genome and the coffee and grape genomes, which did not experience additional duplication events after the ancestral WGT-γ (Figure 2C). In order to determine the age of Ip-α, we used two different phylogenies (Figure 2A). The plastid genome phylogeny supports the monophyly of Aquifoliales as the first diverging clade of campanulids (Magallón et al., 2015); the alternative nuclear genome phylogeny supports *Ilex* in Aquifoliales I as an early branching lineage of lamiids (Zhang et al., 2020b). With the former phylogeny, we estimated the age of the WGD event between 48.75 and 69.63 Ma while, with the latter, divergence was estimated at 49.43 and 70.62 Ma (Figure 2A). Both estimates are consistent with that of Zhang et al., 2020a and validate the age of Ip-α near the origin of *Ilex*, which is estimated between 43 and 89 Ma (Yao et al., 2021).

**Figure 2—figure supplement 1.**
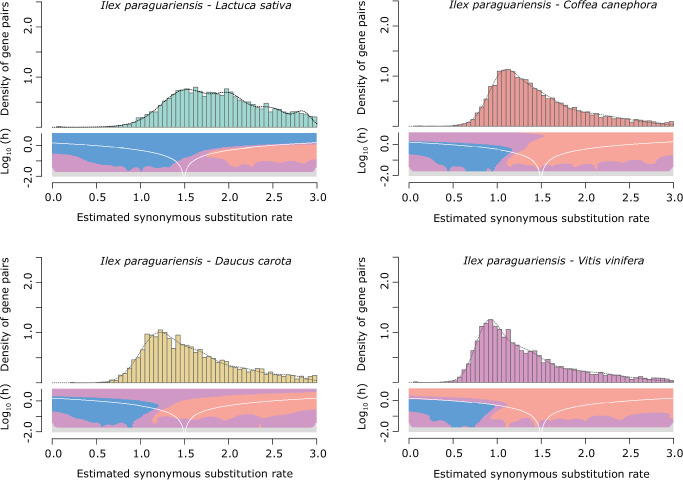
*K*_s_ distributions with Gaussian mixture model and SiZer analyses of *I.* *paraguariensis* and *L. sativa* (green), *D. carota* (yellow), *C. canephora* (red), and *V. vinifera* (purple) orthologues. SiZer maps below histograms identify significant peaks at corresponding *K*_s_ values. Blue represents significant increases in slope, red indicates significant decreases, purple represents no significant slope change, and grey indicates not enough data for the test.

### Convergent evolution of caffeine biosynthesis in YM

**Figure 3.**
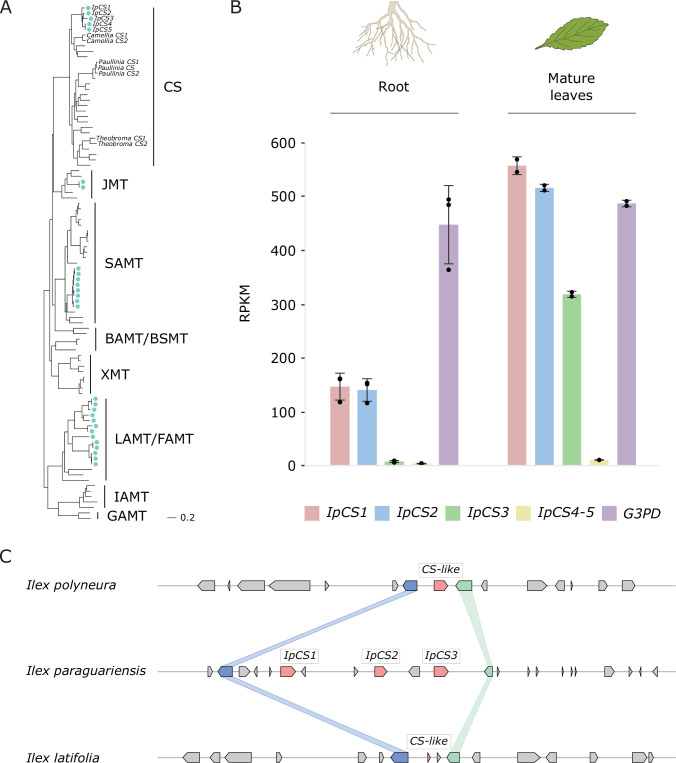
The yerba mate (YM) genome encodes three recently duplicated CS-type SABATH proteins that are expressed in caffeine-producing tissues. (**A**) SABATH gene tree estimate (LnL = −34,265.473) shows the placement of full-length YM proteins (marked by blue-green dots) within clades that have published functions. GAMT, gibberellin MT; IAMT, indole-3-acetic acid MT; LAMT/FAMT, loganic/farnesoic acid MT; BAMT/BSMT, benzoic/salicylic acid MT; XMT, xanthine alkaloid MT used for caffeine biosynthesis in *Coffea* and *Citrus*; SAMT, salicylic acid MT; JMT, jasmonic acid MT; CS, caffeine synthase in *Theobroma*, *Camellia,* and *Paullinia*. Accession numbers for all sequences are provided in Figure 3—source data 1. (**B**) Gene expression analysis of IpCS1–5 in root (*n* = 3) and mature leaves (*n* = 2) as indicated by the relative abundance of YM transcriptome reads mapped to the IpCS1–5 transcripts. RPKM, reads per kilobase per million mapped reads. Error bars indicate standard deviation from the mean. Housekeeping gene: *G3PD*, glyceraldehyde-3-phosphate dehydrogenase. (**C**) Synteny-based analysis of the CS genomic region for *I. paraguariensis*, *I. polyneura*, and *I. latifolia*. Figure 3—source data 1.Accession numbers of SABATH sequences used for phylogenetic analysis in Figure 3.

In order to determine the genes and biochemical pathway responsible for caffeine biosynthesis in YM, we used bioinformatic analyses to identify SABATH enzyme family members in the genome (Huang et al., 2016; Kato et al., 1996; Uefuji et al., 2003). There appear to be 28 full-length SABATH genes in YM that encode members of the functionally diverse clades of the family, including SAMT (Ross et al., 1999) and JMT (Seo et al., 2001), among others (Figure 3A). Our phylogenetic analysis showed that although the YM genome does not appear to encode XMT-type caffeine-producing enzymes like *Coffea* and *Citrus*, it does contain three recently and tandemly duplicated genes that encode CS-type enzymes, IpCS1, IpCS2, and IpCS3 (Figure 3A, C, Appendix 2). The duplicated IpCS1–3 are 86–91% identical at the amino acid level and are expressed at highest levels in caffeine-accumulating tissue (Figure 3B). IpCS1–3 also appear to be of recent origin, since non-caffeine accumulating *Ilex* species only have a single gene or gene fragment in the syntenic region (Figure 3C). In *Camellia*, *Theobroma*, and *Paullinia*, recent duplications of the CS-type enzymes responsible for the successive steps of xanthine alkaloid methylation have also independently occurred (Figure 3A; Huang et al., 2016; O’Donnell et al., 2021). Two other YM genes encode IpCS4 and 5, but these are not syntenic with IpCS1–3 and are not highly expressed in any tissues studied (Figure 3B); therefore, we did not characterize them further.

**Figure 4.**
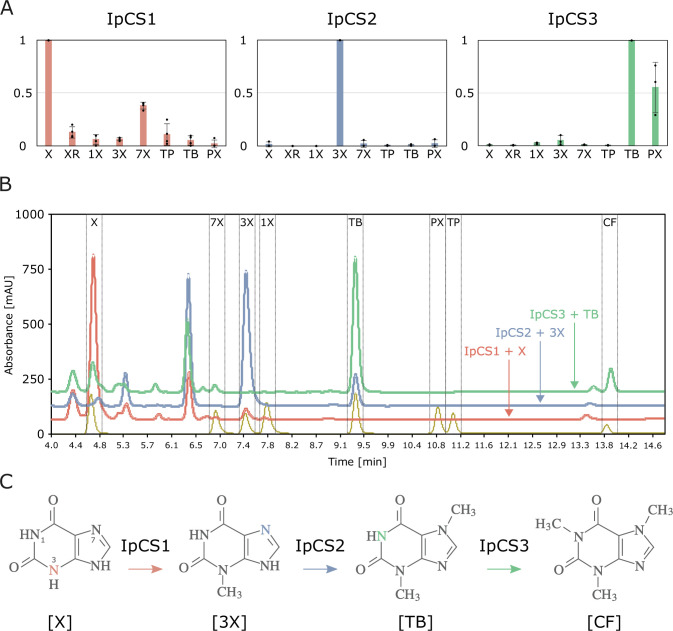
SABATH enzymes have evolved to catalyse the biosynthesis of caffeine in yerba mate. (**A**) Relative enzyme activitiy of IpCS1 (*n* = 4), IpCS2 (*n* = 3), and IpCS3 (*n* = 3) SABATH enzymes with eight xanthine alkaloid substrates. (**B**) High-performance liquid chromatography (HPLC) traces showing products formed by three encoded caffeine synthase (CS)-type enzymes. Absorbance at 254 nm is shown. (**C**) Proposed biosynthetic pathway for caffeine in yerba mate. X, xanthine; XR, xanthosine; 1X, 1-methylxanthine; 3X, 3-methylxanthine; 7X, 7-methylxanthine; TP, theophylline; TB, theobromine; PX, paraxanthine. Coloured atoms and arrows indicate atoms that act as methyl acceptors for a given reaction.

To investigate the biochemical activities of the enzymes encoded by the three CS-type genes, we cloned them into bacterial expression vectors and determined heterologous protein functions. One enzyme, IpCS1, appears to primarily methylate X to catalyse the formation of 3X (Figure 4). A second enzyme, IpCS2, shows activity only with 3X to produce TB, while a third enzyme, IpCS3, exhibits a preference to methylate TB to form CF (Figure 4). Thus, collectively, these three enzymes appear capable of catalysing a complete pathway from xanthine to caffeine. The apparent *K*_M_ for the preferred substrates of all three enzymes ranges from 85 to 197 μM, and the *k*_cat_/*K*_M_ estimates are comparable to those determined for other caffeine biosynthetic enzymes (O’Donnell et al., 2021; Table 4, Figure 4—figure supplement 1). Further evidence for this biosynthetic pathway has been reported by ^14^C xanthine tracer studies in young leaf segments of *I. paraguariensis* that showed radioactivity in 3X and TB in addition to CF (Yin et al., 2015). A pathway from X→3X→TB→CF has also been reported for *Theobroma* and *Paullinia* using CS-type SABATH enzymes (Huang et al., 2016). Like Huang et al., 2016, this represents another departure from the long-assumed pathway to caffeine biosynthesis (XR→7X→TB→CF) as reported in coffee and tea (Figure 1). This instance in *Ilex* is particularly notable since YM is an Asterid, like coffee and tea. The fact that *Ilex*, *Theobroma*, and *Paullinia* convergently recruited CS genes that independently duplicated and evolved to encode enzymes with similar substrate preferences to catalyse a common pathway to caffeine, in spite of their divergence more than 100 Ma (Yang et al., 2020), is remarkable and suggests a high degree of genetic constraint governing the repeated origin of this trait.

**Figure 4—figure supplement 1.**
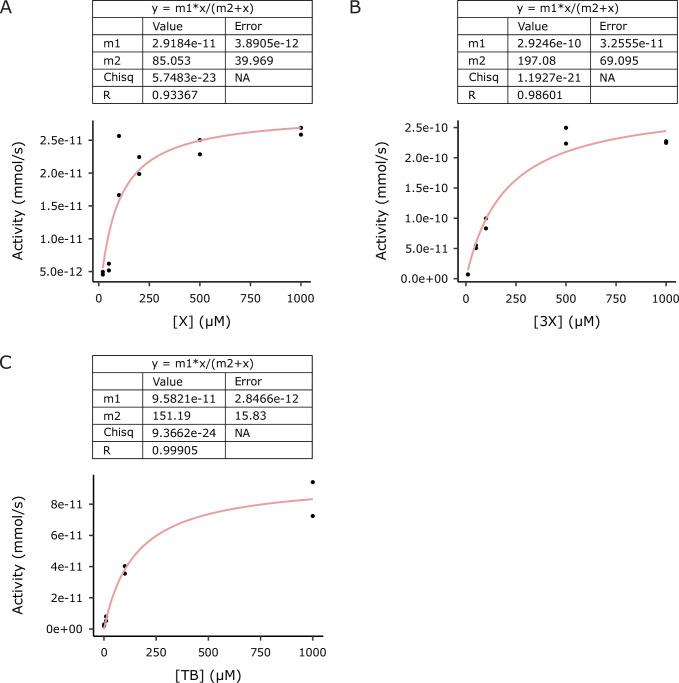
Michaelis–Menten curves used to estimate kinetic parameters for (**A**) IpCS1, (**B**) IpCS2, and (**C**) IpCS3 (*n* = 2). *m*1 is the estimate for *V*_max_ and *m*2 is the estimate for *K*_M_. X, xanthine; 3X, 3-methylxanthine; TB, theobromine.

**Table 4.** Apparent enzyme kinetic parameter estimates for yerba mate caffeine biosynthetic enzymes with selected substrates.

Enzyme (substrate)	*K*_M_ (μM)	*k*_cat_ (1/s)	*k*_cat_/*K*_M_ (s^–1^ M^–1^)
IpCS1 (X)	85.05	0.0009	10.11
IpCS2 (3X)	197.08	0.0031	15.77
IpCS3 (TB)	151.19	0.0029	19.36

While the substrate preferences shown in Figure 4 suggest pathway flux from X→3X→TB→CF, IpCS1 also shows secondary activity with 7X to produce TB and IpCS3 can catalyse the formation of CF from paraxanthine (PX) (Figure 4A). Thus, flux through other branches of the xanthine alkaloid biosynthetic network (Figure 1) cannot be excluded. However, it is not clear how 7X or PX would be produced in planta since none of the three enzymes studied here is capable of their formation; therefore, these secondary activities may not be physiologically relevant. In addition, it has been proposed that TP may also be a precursor to caffeine biosynthesis in *I. paraguariensis* based on radioisotopic feeding studies (Yin et al., 2015), although its levels in plant tissues are 30–160 times lower than TB (Negrin et al., 2019). Our in vitro enzyme assays provide no experimental evidence for that biosynthetic route; however, it is possible that additional MT enzymes from the SABATH (or other) gene family not characterized in this study may perform such reactions. Alternatively, if the exogenously supplied TP was first catabolized to 3X in YM tissues, then the caffeine detected previously (Yin et al., 2015) could have been synthesized via the route described above for IpCS2 + IpCS3 (Figure 4).

### The caffeine biosynthetic pathway in YM evolved from ancestral networks with different inferred flux

**Figure 5.**
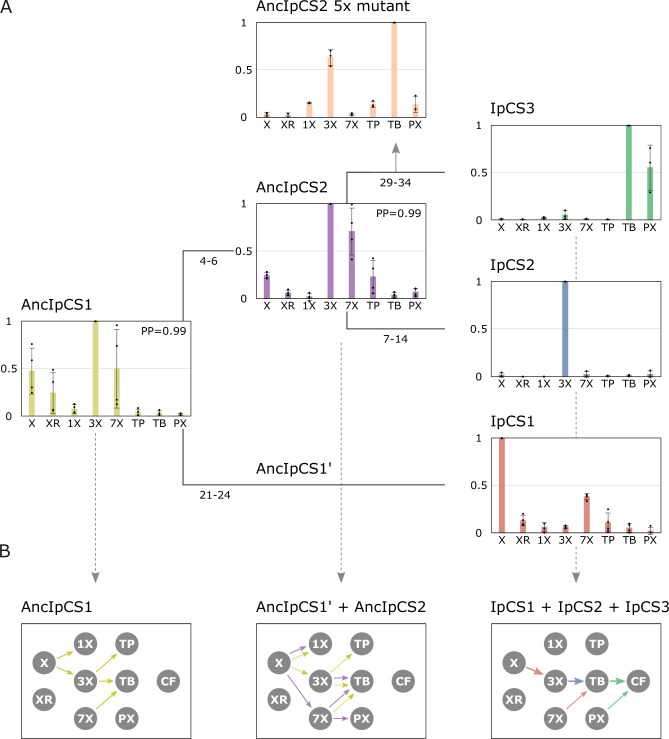
Ancestral sequence resurrection reveals ancestral xanthine alkaloid pathway flux. (**A**) Simpliﬁed evolutionary history of three yerba mate (YM) xanthine alkaloid-methylating enzymes and their two ancestors, AncIpCS1 and AncIPCS2. Average site-speciﬁc posterior probabilities (PP) for each ancestral enzyme estimate are provided. Numbers below each branch of the phylogeny represents the number of amino acid replacements between each enzyme shown. These two ancestral relative activity charts (*n* = 4) show the averaged activities of two allelic variants of each enzyme. Relative substrate preference is also shown for the AncIPCS2 mutant enzyme (*n* = 3) in which five amino acid positions, A22G, R23C, T25S, H221N, and Y265C, that are inferred to have been replaced during the evolution of IpCS3, were changed. (**B**) Inferred pathway ﬂux is shown for the antecedent pathways that could have been catalysed by the ancestral or modern-day combinations of enzymes that would have existed at three time points in the history of the enzyme lineage. Arrows linking metabolites are coloured according to the activities detected from each enzyme shown in panel A. Dotted arrows are shown for AncIpCS1’ because it is unknown what characteristics it would possess; it is assumed that it would have at least catalysed the formation of 3X from X since both its ancestor and descendant enzyme do so. X, xanthine; XR, xanthosine; 1X, 1-methylxanthine; 3X, 3-methylxanthine; 7X, 7-methylxanthine; TP, theophylline; TB, theobromine; PX, paraxanthine.

Caffeine is produced within only one small lineage of *Ilex* that diverged and experienced CS gene duplication (Figure 3) within the last 11 million years (Negrin et al., 2019; Yao et al., 2021) which indicates that the pathway has only recently evolved. The nature by which novel multistep biochemical pathways evolve is a central question in biology (Noda-Garcia et al., 2018). To investigate the caffeine pathway origin in YM, we used Ancestral Sequence Reconstruction (Dean and Thornton, 2007; Thornton, 2004) to study AncIpCS1 and AncIpCS2, the ancestors of the three modern-day enzymes implicated in caffeine biosynthesis in YM (Figure 5, Figure 5—figure supplements 1–4). The ancestral enzyme, AncIpCS1, which gave rise to all three modern-day YM enzymes, exhibits highest relative activity with X, 3X, and 7X (Figure 5A). Methylation of 7X by AncIpCS1 occurred at the N3 position resulting in TB synthesis, whereas xanthine methylation occurred at either the N1 or N3 position to form 1X and 3X, respectively (Figure 5B, Figure 5—figure supplement 5A). AncIpCS1 was capable of methylation of 3X at N1 to produce TP, while methylation at the N7 position led to TB formation (Figure 5B, Figure 5—figure supplement 5A). These data demonstrate that, although AncIpCS1 could not produce caffeine, it could methylate xanthine alkaloids at 3 different positions of the planar heterocyclic ring structures and this combination of activities would have allowed for the ancestor of YM to produce a cocktail of 1X, 3X, TP, and TB by flux through multiple branches of the xanthine alkaloid biosynthetic network with a single enzyme (Figure 5B).

**Figure 5—figure supplement 1.**
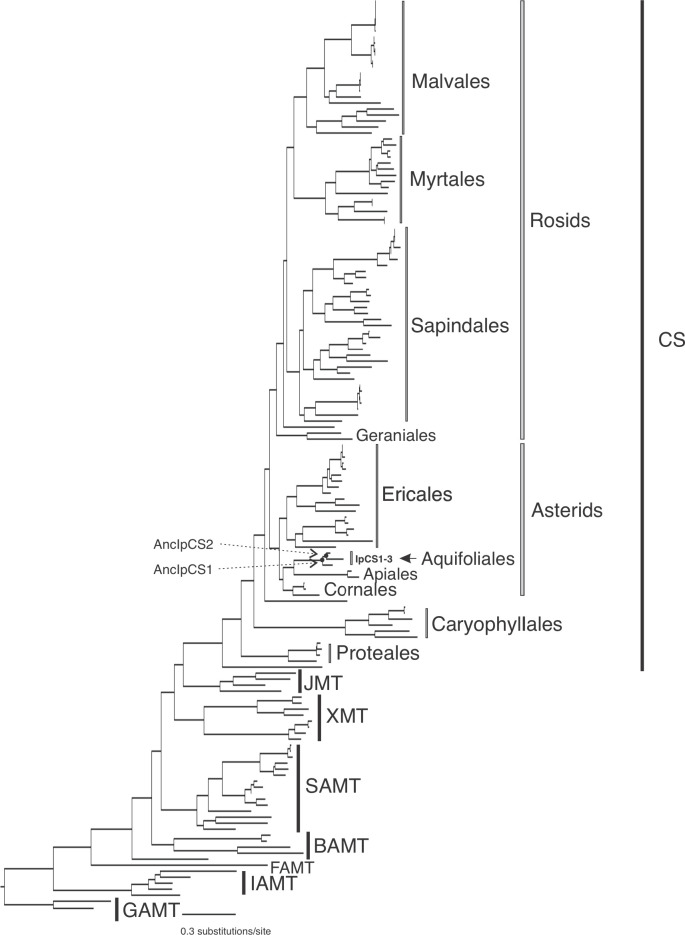
SABATH enzyme family phylogenetic tree used for obtaining ancestral sequence estimates for the clade including IpCS1–3 of Aquifoliales (log-likelihood = −46,631.672). Clades of enzymes for which at least one sequence has been functionally characterized are labelled. GAMT, gibberellin MT; IAMT, indole-3-acetic acid MT; FAMT, farnesoic acid MT; BAMT, benzoic acid MT; XMT, xanthine alkaloid MT used for caffeine biosynthesis in *Coffea* and *Citrus*; SAMT, salicylic acid MT; JMT, jasmonic acid MT; CS, caffeine synthase in *Theobroma*, *Camellia*, and *Paullinia*. Within the CS clade, the orders of rosids and asterids are labelled to show interrelationships.

**Figure 5—figure supplement 2.**
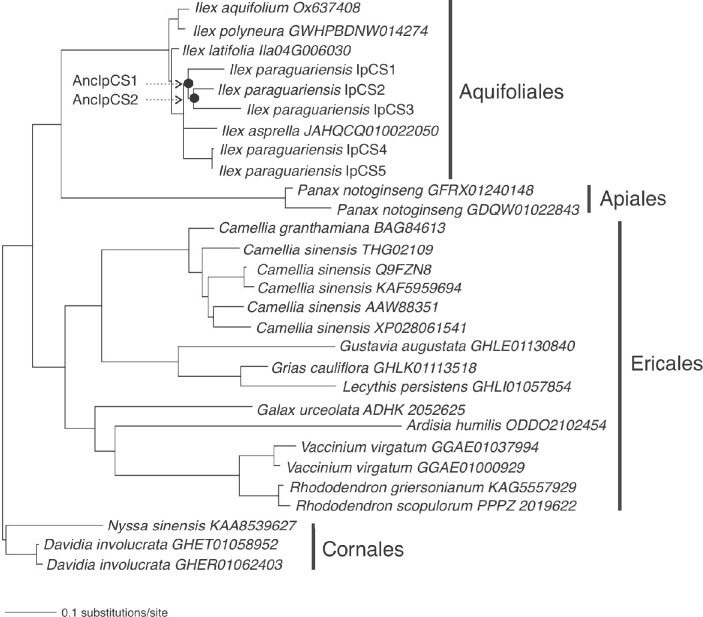
Caffeine synthase enzyme family phylogenetic tree used for obtaining alternative ancestral sequence estimates for AncIpCS1 and 2 (log-likelihood = −7032.8928).

**Figure 5—figure supplement 3.**
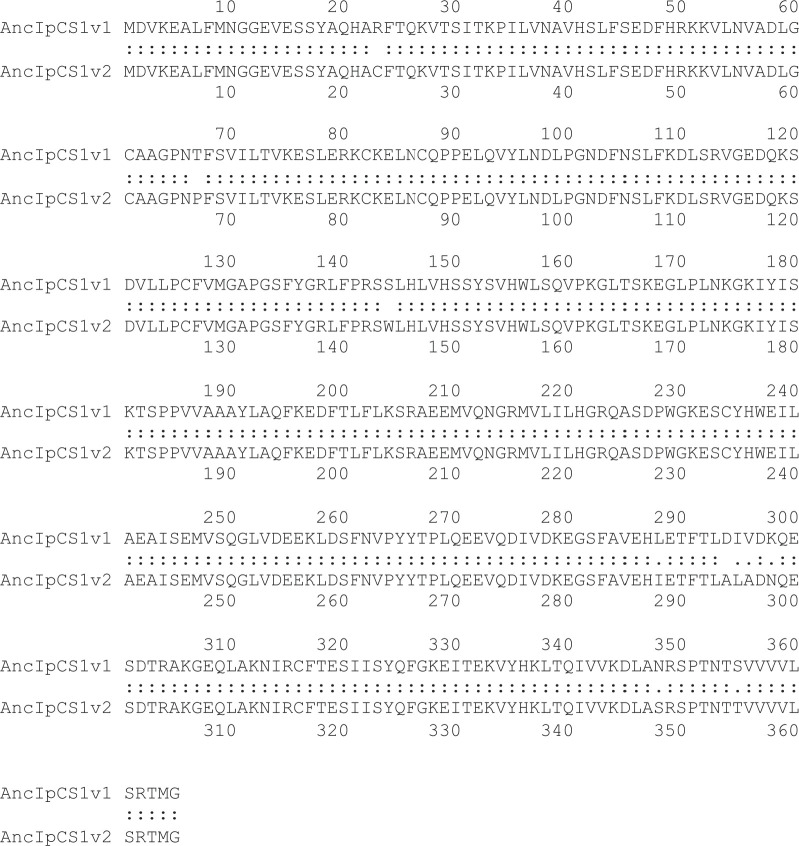
Alignment of the two estimated amino acid sequences for AncIpCS1 that were biochemically characterized in Figure 5.

**Figure 5—figure supplement 4.**
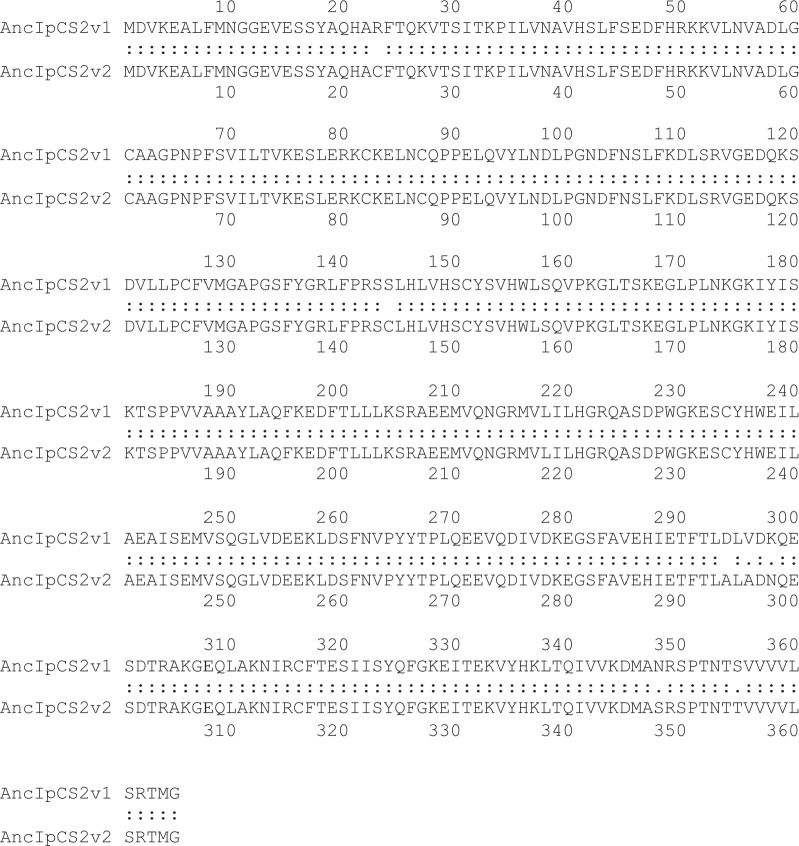
Alignment of the two estimated amino acid sequences for AncIpCS2 that were biochemically characterized in Figure 5.

**Figure 5—figure supplement 5.**
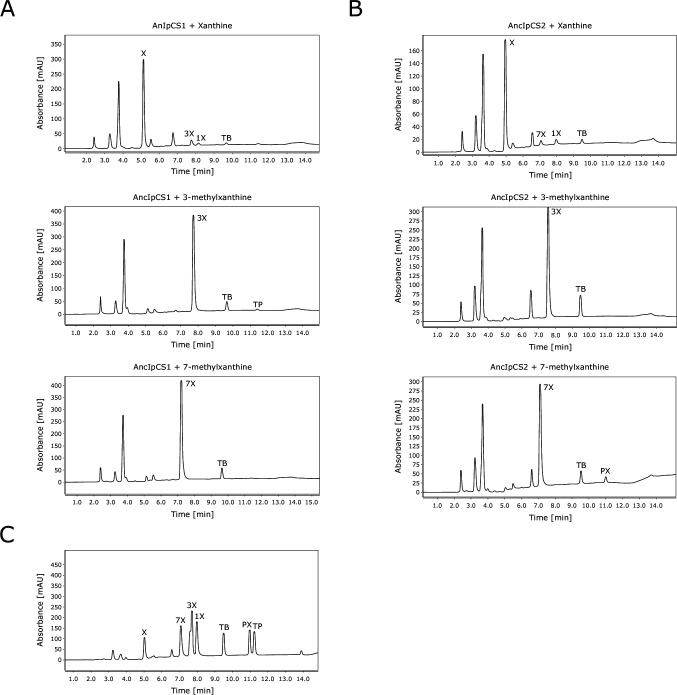
High-performance liquid chromatography (HPLC) traces for xanthine alkaloid products formed by ancestral *Ilex* caffeine synthase (CS) enzymes. (**A**) Chromatograms for AncIpCS1 assayed with three substrates. (**B**) Chromatograms for AncIpCS2 assayed with three substrates. (**C**) Chromatogram for authentic standards. X, xanthine; XR, xanthosine; 1X, 1-methylxanthine; 3X, 3-methylxanthine; 7X, 7-methylxanthine; TP, theophylline, TB, theobromine; PX, paraxanthine. Absorbance at 254 nm is shown.

After gene duplication of AncIpCS1, one daughter enzyme ultimately gave rise to IpCS1, which exhibits preference to methylate xanthine to produce 3X (Figure 5A). The other daughter enzyme, AncIpCS2, appears to have maintained highest activity with X, 3X, and 7X like AncIpCS1 (Figure 5A). However, unlike its ancestor, AncIpCS1, AncIpCS2 evolved high relative activity with 7X to produce not just TB, but also PX by methylation at the N1 position (Figure 5A, Figure 5—figure supplement 5B). AncIpCS2 retained the ancestral activity of AncIpCS1 with xanthine to produce 1X, but also evolved the ability to methylate X at the N7 position (Figure 5B, Figure 5—figure supplement 5B). This enzyme also retained ancestral activity with 3X to produce only TB by N7 methylation but lost the ability to methylate 3X at the N1 position to form TP. These activities of AncIpCS2 would have allowed for ancestral flux to produce 1X, 7X, TB, and PX but not caffeine. Because a YM ancestor could have possessed both AncIpCS2 and a descendant of AncIpCS1, AncIpCS1’ (Figure 5B), additional pathway flux is possible. If AncIpCS1’ retained activities of its ancestor, AncIpCS1, then the ancestral *Ilex* species could have also produced 3X and TP making for an even more diverse array of xanthine alkaloids in its tissues (Figure 5B). It has been shown that the xanthine alkaloids, 1X, 3X, and TP, can bind to modern-day rat adenosine receptors (Daly et al., 1983). Therefore, if these molecules were to accumulate in ancestral *Ilex* tissues, they could have conferred a selective advantage which would likely result in retention of the ancient genes. Ultimately, once gene duplication led to the generation of the three modern-day CS-type enzymes in YM, pathway flux could be channelled away from intermediates like 1X and TP such that the modern-day pathway to caffeine evolved (Figure 5B). Not only did the modern-day CS enzymes of YM evolve to catalyse a pathway from X>3X>TB>CF from ancestral biosynthetic networks of different products, *Theobroma* and *Paullinia* also independently evolved enzymes with similar properties (Huang et al., 2016). And, they did so from ancestral pathways that, like YM, had alternative ancestral fluxes (O’Donnell et al., 2021). While it could be due to chance alone that all three lineages converged to catalyse a similar pathway from differing ancestral networks, it is also possible that it was advantageous to specialize for flux to TB via X and 3X because either it is more enzymatically favourable or these intermediates have greater adaptive value than other structural isomers.

### Protein crystal structure of IpCS3 reveals convergent structural basis for methylation of theobromine to form caffeine

**Figure 6.**
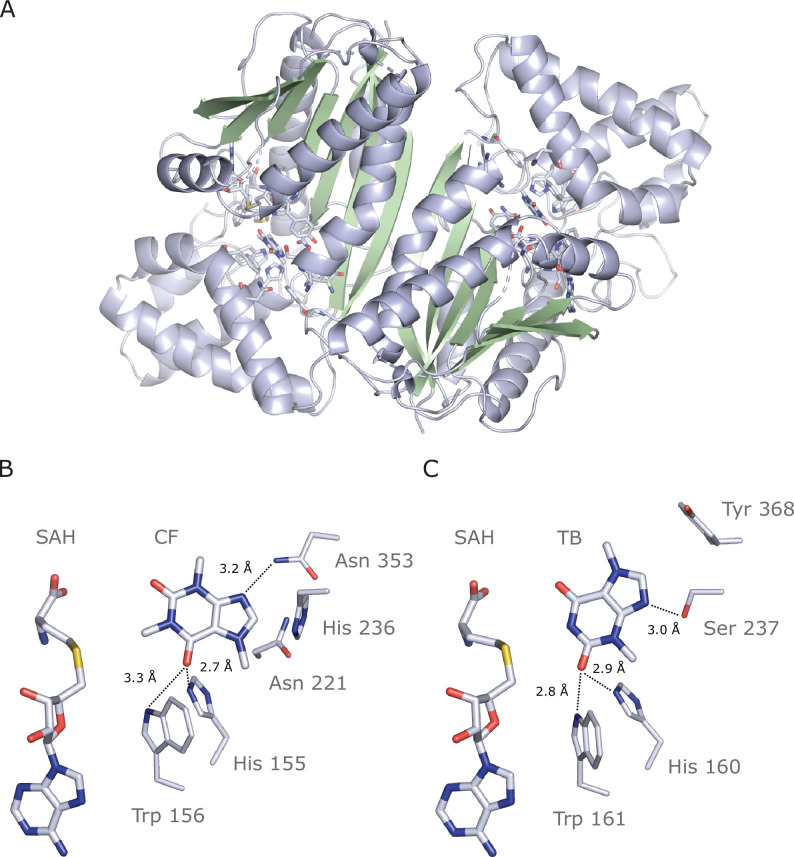
Crystal structure of IpCS3 in complex with caffeine (CF) and *S*-adenosyl-homocysteine (SAH) and comparison with the active site of *Coffea canephora* DXMT. (**A**) Overview of the crystal structure of IpCS3 (PDB ID: 8UZD) depicting the active site of the enzyme in complex with CF and SAH. (**B**) Relevant residues in IpCS3 for ligand recognition. (**C**) Relevant residues in CcDXMT (PDB ID: 2EFJ) for ligand recognition. Protein residues are displayed as lines with carbon atoms coloured in bluewhite while small molecules – CF, theobromine (TB), and SAH – are drawn as sticks. Colour code for the rest of the atoms: nitrogen (blue), oxygen (red), and sulphur (yellow). Hydrogen bond interactions are indicated as black dotted lines.

We successfully crystallized and determined the 2.7 Å resolution structure of IpCS3 (PDB ID: 8UZD), that converts TB into CF. This enzyme crystallizes as a holo-homodimer, bound to both of its reaction products: *S*-adenosyl-homocysteine (SAH) and caffeine (Figure 6A, Table 5). As is typical for the SABATH family of methyltransferases, IpCS3 exhibits a Rossman-like fold composed of seven β-strands surrounded by five α-helices which bind the methyl-donor *S*-adenosyl-L-methionine (SAM), as well as an α-helical cap which binds the methyl-acceptor theobromine (McCarthy and McCarthy, 2007; Petronikolou et al., 2018; Zhao et al., 2008; Zubieta et al., 2003). This structural information of the enzyme bound to both of its products, SAH and caffeine, facilitates an in-depth comparison of the active site structures of the caffeine-producing CS-type enzyme found in *Ilex* to the XMT-type enzyme in *Coffea canephora* (McCarthy and McCarthy, 2007) (CcDXMT) to determine the extent to which convergence of physicochemical properties of the active site has allowed for independent specialization for theobromine methylation by the paralogous SABATH enzymes. Although the IpCS3 structure was obtained in complex with its product, caffeine (Figure 6—figure supplement 1), it can be assumed that the binding mode is conserved for its precursor, theobromine. Indeed, our computational modelling of theobromine in the active site of IpCS3 predicts it to be oriented as we have discerned from the diffraction data (Figure 6—figure supplement 2). Thus, in the following comparisons, the atomic numbering for the theobromine precursor will be used to facilitate comparison to the CcDXMT structure.

**Figure 6—figure supplement 1.**
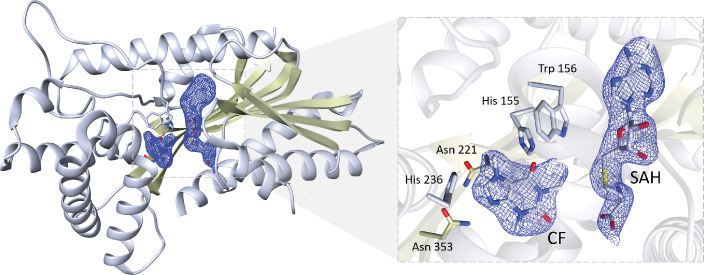
Crystal structure of IpCS3 displaying a difference Fourier map (Fo − Fc) contoured to 2.0 σ (blue) showing bound SAH and CF. Relevant residues in IpCS3 for ligand recognition are displayed as lines with carbon atoms coloured in grey, while small molecules – caffeine (CF) and *S*-adenosyl-homocysteine (SAH) – are drawn as sticks and labelled. Colour code for the rest of the atoms: nitrogen (blue), oxygen (red), and sulphur (yellow).

**Figure 6—figure supplement 2.**
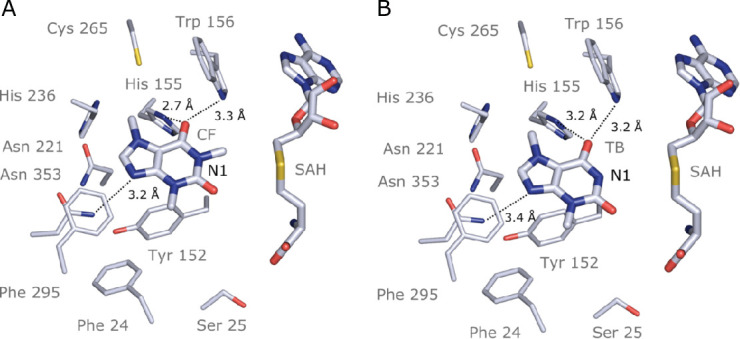
Theobromine and caffeine are oriented the same way in the active site of IpCS3. (**A**) IpCS3–CF complex (PDB ID: 8T2G). (**B**) IpCS3–TB complex (docking model). Protein residues are displayed as lines with carbon atoms coloured in bluewhite while small molecules – theobromine (TB), caffeine (CF), and *S*-adenosyl-homocysteine (SAH) – are drawn as sticks. Colour code for the rest of the atoms: nitrogen (blue), oxygen (red), and sulphur (yellow). Hydrogen bond interactions are indicated as dotted lines.

**Figure 6—figure supplement 3.**
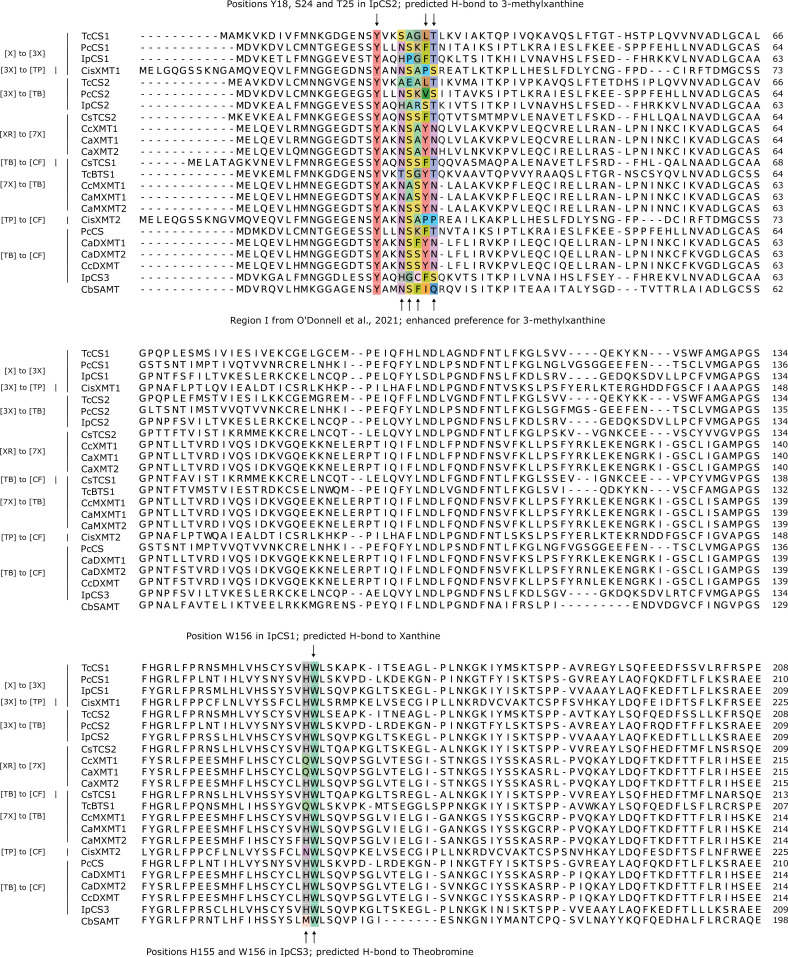
Comparative amino acid alignment of xanthine methyltransferase (XMT) and caffeine synthase (CS) sequences (1–209 of IpCS1) shows convergent changes predicted to participate in substrate binding and promote methylation preference switches. Accession numbers are as follows: TcCS1 (EOY17874), PcCS1 (EC766748), IpCS1 (CAK9135737), CisXMT1 (KDO50937), TcCS2 (EOY17880), PcCS2 (EC778019), IpCS2 (CAK9135740), CsTCS2 (AB031281), CcXMT1 (JX978518), CaXMT1 (AB048793), CaXMT2 (AB084127), CsTCS1 (AB031280), TcBTS1 (AB096699), CcMXMT1 (JX978517), CaMXMT1 (AB048794), CaMXMT2 (AB084126), CisXMT2 (XP_006469448), PcCS (BK008796), CaDXMT1 (AB084125), CaDXMT2 (KJ577793), CcDXMT (JX978516), IpCS3 (CAK9135742), and CbSAMT (AAF00108).

**Figure 6—figure supplement 4.**
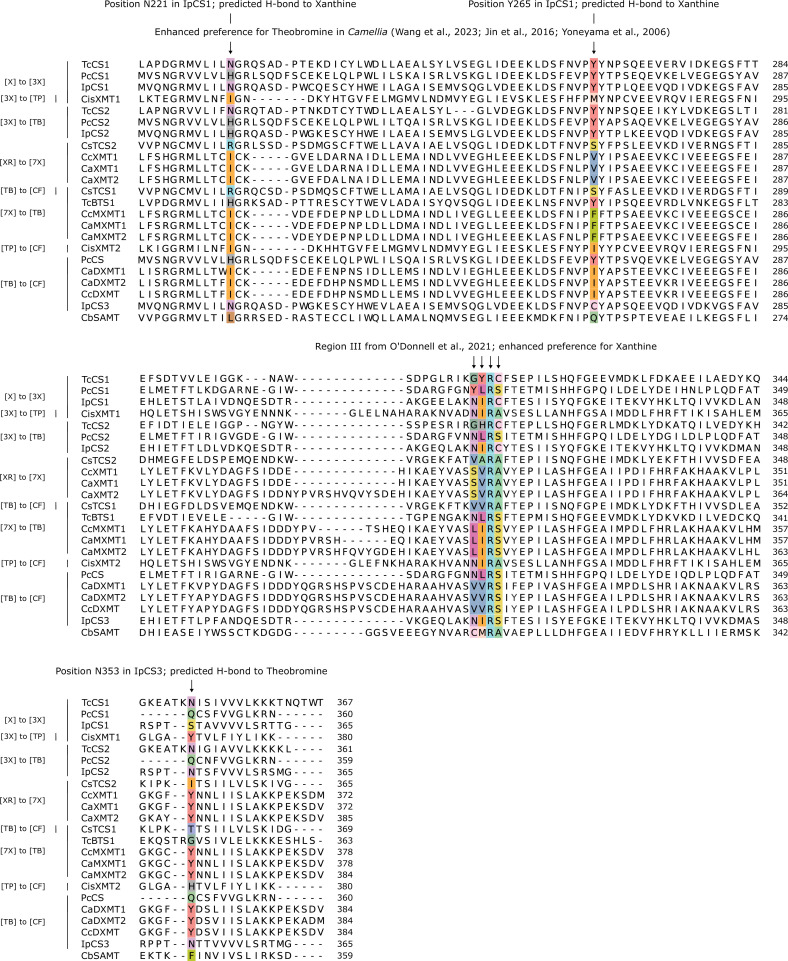
Comparative amino acid alignment of xanthine methyltransferase (XMT) and caffeine synthase (CS) sequences (210–365 of IpCS1) shows convergent changes predicted to participate in substrate binding and promote methylation preference switches. Accession numbers are as follows: TcCS1 (EOY17874), PcCS1 (EC766748), IpCS1 (CAK9135737), CisXMT1 (KDO50937), TcCS2 (EOY17880), PcCS2 (EC778019), IpCS2 (CAK9135740), CsTCS2 (AB031281), CcXMT1 (JX978518), CaXMT1 (AB048793), CaXMT2 (AB084127), CsTCS1 (AB031280), TcBTS1 (AB096699), CcMXMT1 (JX978517), CaMXMT1 (AB048794), CaMXMT2 (AB084126), CisXMT2 (XP_006469448), PcCS (BK008796), CaDXMT1 (AB084125), CaDXMT2 (KJ577793), CcDXMT (JX978516), IpCS3 (CAK9135742), and CbSAMT (AAF00108).

**Table 5.** Data collection and refinement statistics of IpCS3 structure bound to *S*-adenosyl-homocysteine (SAH) and caffeine.

IpCS3 in complex with SAH and caffeine	
**PDB**	8UZD
**Data collection**	
Wavelength (Å)	0.9786
Resolution (Å)	2.72
Resolution range^a*^	37.00–2.72
	(2.82–2.72)
Space group	P 4_1_ 2_1_ 2
Cell dimensions	
*a*, *b*, *c* (Å)	82.67, 82.67, 226.09
*α*, *β*, *γ* (°)	90.00, 90.00, 90.00
Total reflections	43,818
Unique reflections	21,910
Multiplicity^a*^	2.0 (2.0)
Completeness (%)^a*^	99.89 (100.00)
*<I*/σ*I>*^a^	25.79 (2.87)
*R*_merge_^a,b†^* (%)	0.0223 (0.2168)
*R*_meas_ (%)^a*^	0.0315 (0.3066)
CC_1/2_^a*^	0.999 (0.878)
**Refinement**	
Resolution (Å)	2.72
No. reflections	21,909
*R*_work_^c ‡^/*R*_free_^d §^	0.194/0.248
No. atoms	
Protein	5,216
CFF + SAH	80
Water	48
*B*-factors	
Protein	63.38
CFF + SAH	84.48
Water	48.19
Bond lengths (Å)	0.004
Bond angles (°)	1.112

* ^a^Numbers in parentheses refer to the highest resolution shell.

† ^b^*R*_merge_ = Σ|*I_i_* − <*I_i_*>|/Σ*I_i_*, where *I_i_* = the intensity of the *i*th reflection and <*I_i_*> = mean intensity.

‡ ^c^*R*_work_ = Σ|*F*_o_ − *F*_c_|/Σ|*F*_o_|, where *F*_o_ and *F*_c_ are the observed and calculated structure factors, respectively.

§ ^d^*R*_free_ was calculated as for *R*_work_, but on a test set comprising 5% of the data excluded from refinement.

In both CcDXMT and IpCS3, there are several conserved residues, shared by nearly all SABATH enzymes (Figure 6—figure supplements 3 and 4), that form the active site pocket and appear to play important roles in binding many different substrates (Petronikolou et al., 2018; Zubieta et al., 2003). His160 and Trp161 in CcDXMT are in the same relative positions as His155 and Trp156 in IpCS3 (Figure 6B, C). These residues are ca. 3 Å from TB and participate in H-bonding but to different atoms of the substrate. In CcDXMT, the NE2 of His160 and NE1 of Trp161 form hydrogen bonds to carbonyl O2 of TB when positioned for N1 methylation; yet, in the structure of IpCS3 these corresponding side chain groups form hydrogen bonds to O6 of TB. Despite these two residues being conserved for H-bonding, the substrates are rotated 180° along an axis going through N1 and C4. Thus, the conserved His and Trp residues interact with opposing carbonyls in TB/CF but still position the substrate for N1 methylation (Figure 6B, C).

On the other hand, there are residues that differ between the two enzymes but appear to provide for important substrate interactions. Specifically, in the structure of CcDXMT, the hydroxyl group of Ser237 allows specific hydrogen bonding with N9 to position TB for N1 methylation (Figure 6C). In IpCS3, His236 is found at the homologous position in the structure. Nevertheless, its involvement in H-bonding with N9 is uncertain as the distance between nitrogen atoms is ca. 4 Å. Tyr368 of CcDXMT is found to participate in π–π interactions with the ring structure of TB Lanzarotti et al., 2020; yet in IpCS3, Asn353 is found in the homologous position and the amine forms a hydrogen bond with N9 due to its proximity within 3.2 Å, which is additionally stabilized by Asn221 and His236 (Figure 6B). The caffeine-producing CS-type enzymes found in *Camellia sinensis* (CsTCS1) and *Paullinia cupana* (PcCS), may share the same interaction pattern observed with Asn353 in IpCS3 because the homologous Thr in CsTCS1 or Gln in PcCS could potentially form a hydrogen bond with N9 (Figure 6B, Figure 6—figure supplements 3 and 4). Because the residues in these positions of IpCS3 and CcDXMT differ yet contribute to TB binding, these independent replacements represent convergent structural solutions for N1 methylation of the substrate.

In order to experimentally test for the functional importance of the active site residues identified in the crystal structure of IpCS3 for the evolution of TB methylation preference, we performed site-directed mutagenesis. We chose to mutate five amino acid positions that appear to be important for governing xanthine alkaloid methylation in IpCS3 and other CS-type enzymes (Jin et al., 2016; O’Donnell et al., 2021; Wang et al., 2023; Yoneyama et al., 2006); these included A22G, R23C, T25S, H221N, and Y265C (Figure 6—figure supplements 3 and 4). When we mutated all five amino acid residues simultaneously in AncIpCS2, we found that activity with TB increased dramatically relative to 3X and all other xanthine alkaloid substrates (Figure 5A). Thus, these five sites appear to be crucial for the evolution of TB methylation in the history of the YM lineage and further indicate that convergence of caffeine biosynthesis in different species is a result of amino acid replacements at these sites. The homologous sites to H221N and Y265C in Theacrine synthase from *Camellia assamica* were also shown by mutagenesis to be important for the evolution of trimethyluric acid methylation (Zhang et al., 2020a) thereby providing further support for the functional significance of these positions for xanthine alkaloid binding.

### Computational modelling of IpCS1 and IpCS2 active sites predict convergent substrate-binding residues for xanthine and 3-methylxanthine methylation

**Figure 7.**
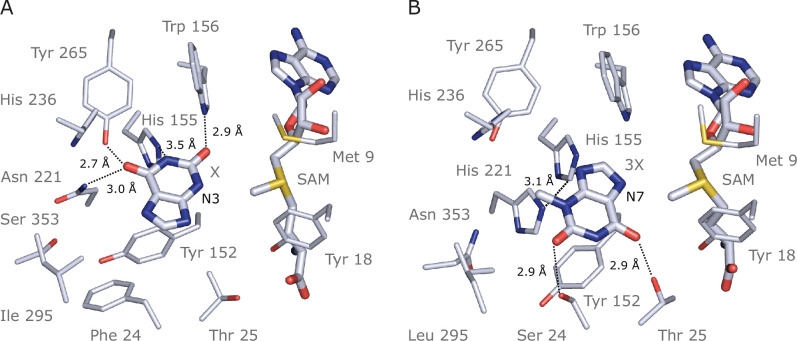
Docking models of xanthine alkaloids in IpCS1 and IpCS2 active sites. (**A**) IpCS1–X complex. (**B**) IpCS2–3X complex. Protein residues are displayed as lines with carbon atoms coloured in bluewhite while small molecules – xanthine (X), 3-methylxanthine (3X), caffeine (CF), paraxanthine (PX), *S*-adenosyl-L-methionine (SAM), and *S*-adenosyl-homocysteine (SAH) – are drawn as sticks. Colour code for the rest of the atoms: nitrogen (blue), oxygen (red), and sulphur (yellow). Hydrogen bond interactions are indicated as black dotted lines.

Previous studies used site-directed mutagenesis of two sequence regions in CS-type caffeine biosynthetic enzymes from *Theobroma* (TcCS1/2) and *Paullinia* (PcCS1/2) to uncover the mutational basis for the convergent evolution of substrate preference switches towards their preferred substrates, X and 3X (O’Donnell et al., 2021). In order to determine whether the same regions were convergently mutated in IpCS1 and IpCS2, and provide binding interactions with X and 3X, respectively, AlphaFold2 (Mirdita et al., 2022) models and subsequent docking studies were performed (Figure 7, Figure 7—figure supplement 1). Modelling of substrate binding in the predicted active sites of IpCS1 and IpCS2 (Figure 7A and B) shows that the preferred substrates have optimal binding orientations that would result in methylation to form the products that were experimentally detected in our assays shown in Figure 4. From our docking simulations, IpCS1 residues W156, N221, and Y265 are positioned for hydrogen bonding with the carbonyl moieties of xanthine to position N3 for methyl transfer from SAM (Figure 7A). Although *Theobroma* and *Paullinia* CS1 enzymes, as well as *Citrus* XMT1, specialized for xanthine methylation also possess W156 and Y265 in the homologous positions (Figure 6—figure supplements 3 and 4), these residues are highly conserved among nearly all angiosperm SABATH enzymes. On the other hand, the homologous position to N221 which is important for IpCS1 did not change concomitantly with the evolution of X preference in either *Theobroma* or *Paullinia* (Figure 6—figure supplements 3 and 4); instead, when *Theobroma* and *Paullinia* ‘region III’ was mutated, activity with X improved (O’Donnell et al., 2021). Because IpCS1 was not mutated in the homologous region III, there appear to be convergent solutions allowing for efficient positioning of X for 3X biosynthesis among these enzymes. In the case of IpCS2, two hydrogen bond donors, S24 and T25, appear to contribute to the positioning of 3X in the active site (Figure 7B). This homologous region was experimentally mutated in *Theobroma* and *Paullinia* CS2 enzymes and improved specialization for 3X methylation in both, although the actual substitutions are different in each case (O’Donnell et al., 2021). Thus, this may represent an additional instance where convergent mutations of the same region lead to specialization for 3X methylation. If crystal structures could be generated for all of these caffeine-producing enzymes in the future, even more detailed insights about active site architecture could be gleaned and would further enhance our understanding of these convergent activities.

**Figure 7—figure supplement 1.**
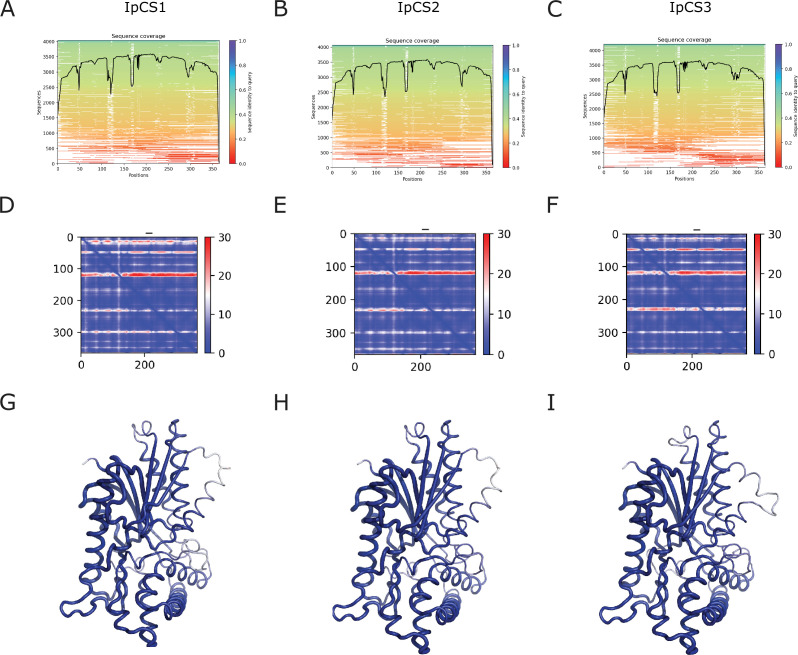
AlphaFold2-ColabFold Model Quality assessment of IpCS1, IpCS2, and IpCS3 models. Sequence coverage of the multiple sequence alignment used for IpCS1 (**A**), IpCS2 (**B**), and IpCS3 (**C**). Alignment error for IpCS1 (**D**), IpCS2 (**E**), and IpCS3 (**F**). pLDDT score of IpCS1 (**G**), IpCS2 (**H**), and IpCS3 (**I**).

### Comparative phylogenomic analyses of caffeine biosynthetic genes reveal historical constraints to convergent gene co-option

**Figure 8.**
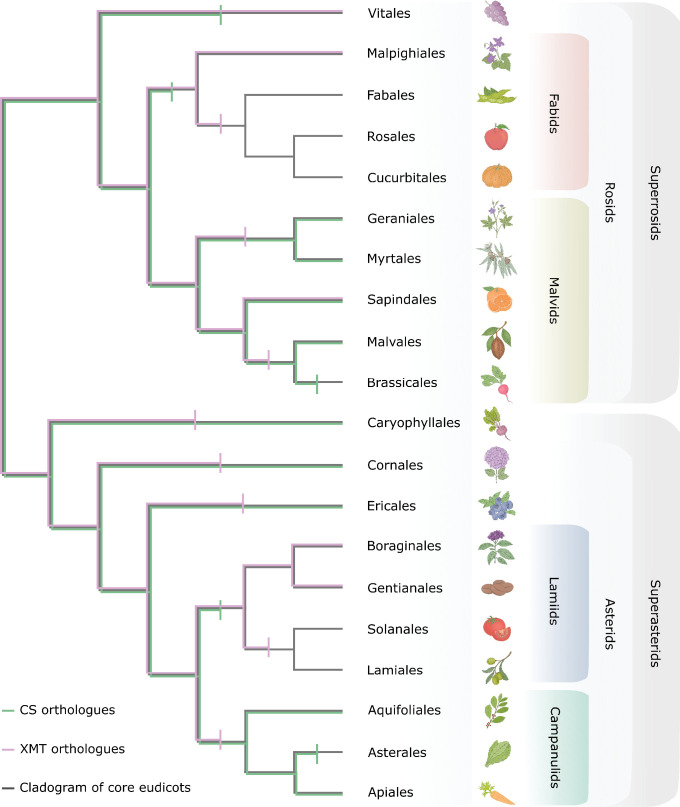
Only CS genes were available for co-option and utilization for xanthine alkaloid biosynthesis in yerba mate whereas coffee only had xanthine methyltransferase (XMT) genes. Both CS- and XMT-type caffeine biosynthetic enzymes were present in the ancestor of core eudicots but numerous apparent losses of one or the other or both has occurred during lineage diversification. Gene loss is represented by vertical bar on relevant branches of the cladogram.

Many nearly ubiquitous specialized metabolites involved in defence, development and floral scent are produced by SABATH enzyme family members that appear to be conserved across diverse angiosperm lineages, such as SAMT that methylates salicylic acid (Dubs et al., 2022) and IAMT that methylates indole-3-acetic acid (Zhao et al., 2008). However, caffeine is sporadically distributed among disparate angiosperm lineages and seems to have only recently evolved by convergence in a few distantly related orders (Huang et al., 2016). Our comparative evolutionary genomic analysis of the CS and XMT syntenic regions across angiosperm (Figure 8) indicates that predicting which SABATH locus a given lineage might co-opt for caffeine biosynthesis is more dependent upon the idiosyncratic history of gene loss than phylogenetic relatedness. For example, in the case of the CS syntenic region used for caffeine biosynthesis in YM and *Theobroma*, *Coffea* lacks a CS orthologue and none can be detected from its genome (Figure 8—figure supplement 1A). Thus, only XMT was historically available for recruitment in *Coffea*. Conversely, YM appears to have lost any vestiges of XMT orthologues known to be responsible for caffeine biosynthesis in *Coffea* and *Citrus* (Figure 8—figure supplement 1B–D). This lack of genomic potential may be seen as an evolutionary constraint to gene recruitment for caffeine biosynthesis in *Coffea* and YM.

**Figure 8—figure supplement 1.**
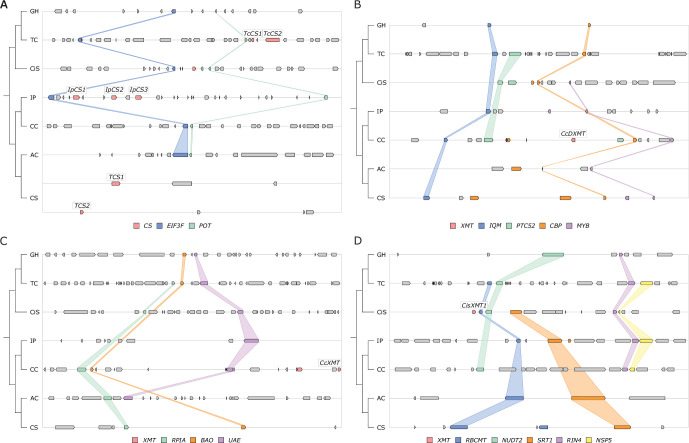
Only CS genes are available for co-option and utilization for xanthine alkaloid biosynthesis in yerba mate. (**A**) Synteny-based analysis of the CS genomic region for seven angiosperm taxa. (**B–D**) Synteny-based analyses of the XMT genomic regions for seven angiosperm taxa. Angiosperm taxa: GH, *Gossypium hirsutum*; TC, *Theobroma cacao*; CiS, *Citrus sinensis*; IP, *Ilex paraguariensis*; CC, *Coffea canephora*; AC, *Actinidia chinensis*; CS, *Camellia sinensis*. Genes: CS, caffeine synthase-type enzyme; EIF3F, eukaryotic translation initiation factor 3 subunit F; POT, proton‐dependent oligopeptide transporter; XMT, xanthine methyltransferase-type enzyme; RPIA, ribose 5-phosphate isomerase A; BAO, beta-amyrin 28-oxidase-like; UAE, UDP-arabinose 4-epimerase 1-like; IQM, IQ domain-containing protein; PTC52, protochlorophyllide-dependent translocon component 52; CBP, calcium-binding protein; MYB, MYB transcription factor; RBCMT, ribulose-1,5 bisphosphate carboxylase/oxygenase large subunit *N*-methyltransferase; NUDT2, nudix hydrolase 2-like, SRT1, NAD-dependent protein deacetylase; RIN4, RPM1 interacting protein 4; NSP5, nitrile specifier protein 5.

A broader phylogenetic perspective on the XMT and CS syntenic regions provides further insight into genomic canalization and allows for predictions about the underlying genetic basis for caffeine biosynthesis in as-of-yet characterized lineages. As shown in Figure 8, several angiosperm lineages have neither XMT nor CS and this may explain why caffeine has apparently never evolved in the large and diverse orders Brassicales, Asterales, Solanales and Lamiales even though it has been shown to be advantageous in transgenic plants (Kim et al., 2011; Kim et al., 2006). In the case of *Cola*, a caffeine-producing genus from Africa (Niemenak et al., 2008), it is predicted to have co-opted CS genes for xanthine alkaloid methylation because the order Malvales to which it belongs appears to have lost XMT orthologues prior to its origin (Figure 8). Tests of this hypothesis await genomic sequences and functional studies of *Cola* enzymes. However, even with a functional XMT or CS enzyme, gene duplication and protein functional diversification appears to be required to assemble a complete pathway to caffeine as shown here for YM. Nonetheless, because molecular clock analyses indicate that the caffeine-producing *Coffea*, *Camellia*, *Citrus*, *Paullinia*, and *Ilex* lineages each originated within only the last 10–20 million years (Buerki et al., 2011; Hamon et al., 2017; Pfeil and Crisp, 2008; Yao et al., 2021; Zan et al., 2023), it suggests that the evolution of novel specialized metabolic pathways like that of caffeine can be relatively rapid.

## Materials and methods

### Plant materials

*I. paraguariensis* A. St.-Hil. var. *paraguariensis*, cv CA 8/74 (INTA-EEA Cerro Azul, Misiones, Argentina) and cv SI-49 (Establecimiento Las Marías S.A.C.I.F.A., Corrientes, Argentina) were used in this study. High productivity, increased tolerance to drought, and ease of propagation with stem cuttings characterize these genotypes (Acevedo et al., 2019; Avico et al., 2023; Tarragó et al., 2012).

### DNA extraction and sequencing

Two DNA extraction and sequencing approaches were combined to improve the accuracy of genome assembly. First, young leaves of cv CA 8/74, preserved in silica-gel, were used to isolate total genomic DNA with the DNeasy Plant Mini Kit (QIAGEN), following the manufacturer’s instructions. Paired-end libraries (with insert sizes of 350 and 550 bp) and mate-pair libraries (with insert sizes of 3, 8, and 12 kbp) were constructed using the Illumina TruSeq DNA Sample Preparation Kit and Illumina Nextera Mate Pair Library Preparation Kit following the kit’s instructions, respectively. The obtained libraries were sequenced on an Illumina HiSeq 1500 platform, generating ~263.2 Gb of raw data. Second, young leaves of cv SI-49, preserved in silica-gel, were used to purify high molecular weight DNA with the Quick-DNA HMW MagBead Kit (Zymo Research), according to the manufacturer’s instructions. Long reads libraries were prepared using Sequel Binding Kit 1.0 (Pacific Biosciences), following the manufacturer’s instructions. The obtained libraries were subsequently sequenced on PacBio Sequel I (Pacific Biosciences) using Sequel Sequencing Kit 1.0 (Pacific Biosciences) and SMRT Cell 1M (Pacific Biosciences), generating ~77.5 Gb of additional raw data.

### Genome assembly and quality assessment

We opted for a pipeline that could take advantage of both short and long sequencing technologies. For the short reads, we applied Trimmomatic v.0.39 (Bolger et al., 2014) to remove adaptor contaminations and filter low-quality reads (reads with mean quality scores ≤25, reads where the quality of the bases at the head or tail was ≤10 and reads with a length ≤30 bp). The resulting clean reads were then corrected using Quake v.0.3 (Kelley et al., 2010). Contig assembly and scaffolding was done using the assembler SOAPdenovo v.2 (Luo et al., 2012) (55-mer size), with the mate-pair reads being used to link contigs into scaffolds. After the assembly, DeconSeq v.0.4.3 (Schmieder and Edwards, 2011) was used to detect and remove sequence contaminants. Contigs and scaffolds clearly belonging to the chloroplast and mitochondria genomes were also discarded. YM transcriptome sequences (Acevedo et al., 2019; Debat et al., 2014; Fay et al., 2018) and public databases KOG (Tatusov et al., 2003) and DEG (Luo et al., 2014) were used to validate the genome assembly. Canu v.2.2 (Koren et al., 2017) was used to perform the self-correction and assembly of the long reads, using the default parameters and stopOnLowCoverage = 20. For both short and long assemblies, we separated the assembly haplotypes (haplotigs) using PurgeHaplotigs (Roach et al., 2018) with the recommended parameter values. Then, we merged both SOAPdenovo v.2 and Canu v.2.2 curated assemblies using Quickmerge v.03 (Chakraborty et al., 2016), where only contigs with minimum overlap of 5000 bp (-ml 5000) were merged and only the contigs greater than 1000 bp (-l 1000) were retained. The resulting scaffolds and contigs were refined further with the gap-filling module in SOAPdenovo v.2 (GapCloser) and SSPACE v.2.1.1 (Boetzer et al., 2011).

### Gene prediction and annotation

First, we masked the genome assembly with RepeatMasker (http://repeatmasker.org/). Then, we predicted the protein- and non-coding genes using Funannotate v.1.8.13 (Palmer and Stajich, 2019) previously training it with the available *I. paraguariensis* RNA-Seq experiments (NCBI projects PRJNA315513, PRJNA375923, and PRJNA251985). Then, Infernal v.1.1.4 (Nawrocki and Eddy, 2013) was employed to improve the prediction of small RNAs and microRNAs, while tRNAScan-SE v.2.0 (Chan and Lowe, 2019) was used to improve the prediction of transfer RNAs. Ribosomal RNAs were predicted using RNAmmer v.1.2 (Lagesen et al., 2007). The TAPIR web server (Bonnet et al., 2010) (http://bioinformatics.psb.ugent.be/webtools/tapir) and the TargetFinder software v.1.7 (Fahlgren and Carrington, 2010) were used to identify miRNA targets. InterProScan v.5.55-88.0 (Jones et al., 2014) and eggNOG-mapper v.2.1.7 (Huerta-Cepas et al., 2019) were employed for the functional assignment of the predicted genes.

### Repeat content estimation

The repeat content was estimated employing Dfam TE Tools v.1.5 (https://github.com/Dfam-consortium/TETools copy archived at Rosen and Gray, 2024). First, we used RepeatModeler v.2.0.3 (Flynn et al., 2020) to build a database with *Ilex* repeat families. Then, we merged that database with Dfam v3.6 (Hubley et al., 2016) and GIRI Repbase ver 20181026 (Jurka et al., 2005). Finally, we ran RepeatMasker on the assembly using the merged database to look for repeat sequences.

### Genome duplication analysis

Rates of synonymous substitution (*K*_s_) between paralogous genes and orthologous genes in *Lactuca sativa*, *Daucus carota*, *I. paraguariensis*, *C. canephora*, and *Vitis vinifera* were determined using CoGe’s tool SynMap (https://genomevolution.org/). Gaussian mixture models were fitted to the resulting *K*_s_ distributions with the mclust R package v.5.0 (Scrucca et al., 2016), and significant peaks were identified using the SiZer R package v.0.1-7 (Chaudhuri and Marron, 2000). To estimate the age of the lineage-specific polyploidization event (Ip-α) in *Ilex*, we considered two different phylogenies (a multiple nuclear genome phylogeny and a plastid genome phylogeny). With the median *K*_s_ value of YM-grape orthologues (~0.89) and the divergence date of the two species (125.64 Ma for the multiple nuclear genome phylogeny and 123.7 Ma for the plastid genome phylogeny), we calculated the number of substitution per synonymous site per year (*r*) for YM (divergence date = *K_s_*/(2 × *r*)). Conforming to the multiple nuclear genome phylogeny, the YM *r* value is 3.54E−9; while for the plastid genome phylogeny, the YM *r* value is 3.59E−9. These *r* values and the SiZer *K*_s_ range of YM paralogues (~0.35–0.5) were then applied to estimate the age of Ip-α. Finally, to determine the syntenic depth ratio between *I. paraguariensis* and *C. canephora* and *V. vinifera*, we employed CoGe’s tool SynFind (https://genomevolution.org/), using a distance cutoff of 10 genes and requiring at least 5 gene pairs per synteny block.

### Gene expression quantitation

First, YM transcriptome reads (PRJNA315513) were mapped to IpCS1–5 transcripts, obtained from the de novo transcriptome assembly and annotation, using BWA (Li and Durbin, 2009). Then, with the number of mapped reads, the abundance of each transcript was calculated, normalized by transcript length and transcriptome size (quantification in RPKM, reads per kilobase per million mapped reads).

### Cloning, mutagenesis, heterologous expression, and purification of enzymes

Two different approaches were used to clone IpCS genes: RT-PCR from leaf tissue and custom gene synthesis. For RT-PCR of IpCS2, cDNA was obtained from 1 µg of RNA from fresh YM leaves using standard procedures and cycling conditions with the following two primers: IpCS2F 5′-ATGGACGTGAAGGAAGCAC-3′ and IpCS2R 5′-CTATCCCATGGTCCTGCTAAG-3′. Following amplification, cDNA was cloned using the pTrcHis TOPO TA Expression Kit (Invitrogen, Carlsbad, CA). Ligation of cDNA into the pTrcHis vector and subsequent transformation into Top10 *E. coli* cells was carried out according to the manufacturer’s protocol. The transformation mixture was incubated overnight at 37°C on LB plates containing 50 µg/ml ampicillin. Colonies were screened by PCR to obtain full-length inserts that were subsequently verified for insert orientation by DNA sequencing. For IpCS1 and 3 and AncIpCS1 and 2, gene sequences were synthesized by GenScript with codons optimized for expression in *E. coli*. Synthesized genes were subcloned from the pUC57 cloning vector into the pET-15b (Novagen) expression vector using 1.5 μg of DNA and NdeI and BamHI in 30 μl reactions. Linear fragments corresponding to the expected sizes were gel purified using the QIAEX II Gel Extraction Kit (QIAGEN Corp) according to the manufacturer’s instructions. Purified DNA fragments were ligated into pET-15b using T4 DNA ligase from New England Biolabs. Ligation products were transformed into Top10 *E. coli* cells using 2 μl of the ligation reaction. Site-directed mutagenesis of AncIpCS2 was carried out using the Agilent QuikChange Lightning Kit (Agilent Technologies Inc, Santa Clara, CA) following the manufacturer’s protocol. Minipreps of positive transformants were obtained using a QIAprep Spin Miniprep Kit (QIAGEN Corp) and 10 ng of each plasmid was used to transform BL21 *E. coli* cells using standard plating and incubation methods.

Induction of His_6_-protein was achieved in 50 ml cell cultures of BL21 (DE3) with IpCS1 and 3 and AncIpCS1 and 2 in pET-15b or Top10 with IpCS2 in pTrcHis with the addition of 1 mM IPTG at 23°C for 6 hr. Purification of the His_6_-tagged protein was achieved by TALON spin columns (Takara Bio) following the manufacturer’s instructions. Bradford assays were used to determine purified protein concentration, and recombinant protein purity was evaluated on sodium dodecyl sulphate–polyacrylamide gel electrophoresis gels.

### Enzyme assays

All enzymes were tested for activity with the eight xanthine alkaloid substrates shown in Figure 1. Radiochemical assays were performed at 24°C for 60 min in 50 µl reactions that included 50 mM Tris–HCl buffer, 0.01 µCi (0.5 µl) ^14^C-labelled SAM, 10–20 µl purified protein, and 1 mM methyl acceptor substrate dissolved in 0.5 M NaOH. Negative controls were composed of the same reagents, except that the methyl acceptor substrate was omitted and 1 µl of 0.5 M NaOH was added instead. Methylated products were extracted in 200 µl ethyl acetate and quantified using a liquid scintillation counter. The highest enzyme activity reached with a specific substrate was set to 1.0 and relative activities with remaining substrates were calculated. Each assay was run at least three times so that mean, plus standard deviation, could be calculated.

### High-performance liquid chromatography

Product identity of enzyme assays was determined using high-performance liquid chromatography (HPLC) on 500 μl scaled-up reactions utilizing all the same reagents as described above except that non-radioactive SAM was used as the methyl donor and reactions were allowed to progress for 4 hr. Whole reactions were filtered through Vivaspin columns (Sartorius) to remove all protein prior to injection in the HPLC. Mixtures were separated by HPLC using a two-solvent system with a 250 mm × 4.6 mm Kinetex 5 μM EVO C18 column (Phenomenex). Solvent A was 99.9% water with 0.1% trifluoroacetic acid and Solvent B was 80% acetonitrile, 19.9% water and 0.1% TFA and a 0–20% gradient was generated over 16 min with a flow rate of 1.0 ml/min. Product identity was determined by comparing retention times and absorbance at 254 and 272 nm of authentic standards. Reactions were compared to negative controls in which no methyl acceptor substrates were added.

### Phylogenetic analyses

In order to accurately determine the orthology of YM SABATH sequences encoded in the genome, we compared them to all previously functionally characterized gene family members in other species. We also included CS and XMT orthologues from the orders of caffeine-producing species (Malvales, Ericales, Gentianales, Sapindales) available in public databases (GenBank, OneKP) as shown in Figure 3. Accession numbers for all sequences are provided in . Alignment of amino acid sequences was achieved using MAFFT v.7.0 (Katoh and Standley, 2013) and employing the auto search strategy to maximize accuracy and speed. A phylogenetic estimate was obtained using FastTree v.2 (Price et al., 2010) assuming the Jones–Taylor–Thorton model of amino acid substitution with a CAT approximation using 20 rate categories. Reliability of individual nodes was estimated from local support values using the Shimodaira–Hasegawa test as implemented in FastTree.

### Ancestral sequence resurrection

In order to obtain accurate ancestral CS protein estimates, we assembled two datasets to assess variation in terms of sampling. The first dataset included 154 sequences including all CS-type enzymes we could retrieve from GenBank and China National Gene Bank as well as representatives of all other functionally characterized clades of SABATH enzymes (Figure 5—figure supplement 1). In this dataset, the only *Ilex* sequences available were IpCS1–3. This dataset resulted in highly confident estimates for AncIpCS1 and AncIpCS2 (average site-specific posterior probabilities >0.99 in both cases). Subsequently, once additional *Ilex* genomes became available, we estimated a second set of ancestral sequences using 29 CS-type enzymes from asterids to assess uncertainty in our initial estimates (Figure 5—figure supplement 2). In this subsequent analysis, highly confident estimates for AncIpCS1 and AncIpCS2 were obtained with average site-specific posterior probabilities >0.99 in both cases (see Figure 5). MAFFT v.7.0 (Katoh and Standley, 2013) was used to align the protein sequences in both datasets using the auto search strategy to maximize accuracy and speed; subsequently, IQTree (Trifinopoulos et al., 2016) was used to obtain trees describing the relationships amongst the aligned sequences for both datasets. For the first set of ancestral sequence estimates, the Jones, Taylor, and Thorton matrix model for amino acid substitution and the Free rate model for among-site rate heterogeneity (Yang, 1995) was determined to be the best fit. For the second dataset, the Q matrix as estimated for plants (Ran et al., 2018) with a gamma model for rate heterogeneity was the preferred model. IQTree estimates ancestral sequences using the empirical Bayesian approach (Trifinopoulos et al., 2016). In order to determine ancestral protein lengths in regions with alignment gaps, we coded each gap for the number of amino acids possessed and used parsimony to determine ancestral residue numbers as in our previous studies (Huang et al., 2016). The estimated sequences were synthesized by Genscript Corp and had codons chosen for optimal protein expression in *Escherichia coli* and were cloned into pET15b for expression and purification using the His_6_ tag. Details of expression were the same as described above for the modern-day enzymes. Although the two separate ancestral sequence estimates are highly similar to one another (>95% identity in both cases), the two AncIpCS1 proteins differ at 10 positions and those for AncIpCS2 differ at seven positions (Figure 5—figure supplements 3 and 4).

### Crystallization, data collection, phasing, and refinement of IpCS3

Initial crystallization screening was performed using the IpCS3 methyltransferase at a concentration of 30 mg/ml incubated with 2 mM TB and 2 mM SAM. Sitting-drop for crystallization screening was set up by equal volume of precipitant and protein solution (0.25:0.25 µl) using a Crystal Gryphon robot (Art Robbins Instruments) and a reservoir volume of 45 µl. Trays were incubated at 9°C. Initial hits were further optimized using the hanging-drop method at 9°C, with 150 µl reservoir solution and 1:1 ratio of precipitant to protein and ligand solution in a 2-µl drop. Attempts to crystallize with SAH or uncleavable SAM analogs and TB to attain a pre methylation structure were unsuccessful given the poor diffraction of these crystals. Therefore, the latter was composed of 33 mg/ml IpCS3 protein concentration, 4 mM TB and 2 mM SAM, and the crystallization condition was optimized to 25% PEG 3350, 0.2 M NH_4_SO_4_, 0.1 M Bis-Tris methane pH 5.5. Square crystals grew over 10 days, but initial X-ray crystallography data revealed a poor electron density for SAH and an electron density in the active site for CF, the product, rather than for TB. Consequently, crystals were grown in the aforementioned condition and subsequently soaked for 4 hr at 9°C in the precipitant solution supplemented with 10 mM SAH and 10 mM TB. The idea was to supply an excess of the expended methyl source and additional TB to convert any existing SAM as we did not have access to caffeine as a reagent. Crystals were transiently soaked in the precipitant solution supplemented with 20% ethylene glycol immediately prior to vitrification by direct immersion into liquid nitrogen. Diffraction data were collected at the Advanced Photon Source (APS) at Argonne National Laboratory Sector-21 via the Life Sciences-Collaborative Access Team (LS-CAT) at beamline 21-ID-G. Diffraction data were indexed, integrated, and scaled using the autoPROC software package (Vonrhein et al., 2011). The structure was solved by molecular replacement using Phaser-MR included in the Phenix software package (Adams et al., 2010), using PDB ID 6LYH structure as the replacement model. The model was subject to rounds of manual building followed by refinement using REFMAC5 (Murshudov et al., 2011), and was manually built in COOT v.0.9.8.3 (Emsley et al., 2010). Crystallographic statistics are listed in Table 5.

### Structure prediction and molecular docking

Protein structures of IpCS enzymes were predicted using the ColabFold implementation of AlphaFold2 (Mirdita et al., 2022) with no template. Diagnostic plots depicting the MSA coverage, alignment error and LDDT are shown in the supplementary information (Figure 7—figure supplement 1). Structures of xanthine alkaloid ligands (X, 3X, and TB) were downloaded from the ChEMBL database Mendez et al., 2019; protonation states were checked by Chemicalize (Swain, 2012) and optimized using the VMD Molefacture plugin (Humphrey et al., 1996). The receptor structures were prepared following the standard AutoDock protocol (Morris et al., 2009) using the prepare_receptor4.py script from AutoDock Tools. All non-polar hydrogens were merged, and Gasteiger charges and atom types were added. The ligand PDBQT was prepared using the prepare_ligand4.py script. The grid size and position were chosen to contain the whole ligand-binding site (including all protein atoms closer than 5 Å from all ligands). For each system, 10 different docking runs were performed. Docking was performed using AutoDock Vina v.1.2.0 (Eberhardt et al., 2021). The docking results were further analysed by visual inspection. Images of the molecules were prepared using the PyMOL molecular graphics system (Schrodinger, 2015).

### Synteny comparisons and phylogenetic distribution of CS and XMT

The presence or absence of CS and XMT genes was determined for orders of plants for which at least one genomic sequence exists, as shown in Figure 8. For those species which do not yet have an available assembly, we used BLAST (Altschul et al., 1990) analyses of GenBank (nr and TSA databases), Phytozome (Goodstein et al., 2012) as well as the OneKP dataset (One Thousand Plant Transcriptomes Initiative, 2019) in China National GeneBank. BLAST combined with subsequent phylogenetic analyses were also used to verify presence/absence of CS- or XMT-type sequences in cases where the syntenic regions did not appear to encode one or the other gene. Comparisons of the CS and XMT syntenic regions were performed using CoGe’s tool GEvo (https://genomevolution.org/).

## Data Availability

The Illumina and PacBio raw sequence data, assembly and annotation were deposited in the European Nucleotide Archive (ENA) under BioProject No. PRJEB65927. An assembly obtained only with the Illumina data was also deposited in ENA under BioProject No. PRJEB36685. The plasmids used to produce proteins are freely available upon request. The atomic coordinates and structure factors have been deposited in the Protein Data Bank, Research Collaboratory for Structural Bioinformatics, Rutgers University, New Brunswick, NJ (http://www.rscb.org) with the accession code 8UZD for the IpCS3 structure bound to caffeine and SAH. The following datasets were generated: VignaleFA 2024Ilex paraguariensis var. paraguariensis genome (Illumina and PacBio)ENAPRJEB65927 VignaleFA 2024Ilex paraguariensis var. paraguariensis genome (Illumina)ENAPRJEB36685 Hernandez GarciaA 2024The structure of IpCS3, a theobromine methyltransferase from Yerba MateRCSB Protein Data Bank8UZD The following previously published datasets were used: FayJV 2016Ilex paraguariensis multiple library de novo transcriptome assemblyENAPRJNA315513 DebatHJ 2014Yerba mate (Ilex paraguariensis St. Hil.) NGS DN Transcriptome assemblyNCBI Sequence Read ArchiveSRP04329310.1371/journal.pone.0109835PMC419971925330175 AcevedoRM 2019RNA-Seq of Ilex paraguariensis: roots and mature leavesNCBI Sequence Read ArchiveSRP110129

## Additional files

MDAR checklist

## Appendix 1

### Non-coding RNAs in yerba mate

#### 1.1 Transfer RNAs

Analysis of transfer RNA (tRNA) family members revealed the presence of 815 tRNA genes including 726 standard tRNAs, 76 pseudo tRNAs, 11 tRNAs with undetermined isotypes, and 2 possible suppressor tRNAs (Appendix 1—table 1). No selenocysteine tRNA was found in the yerba mate genome, which is consistent with the results of other tRNA analyses carried out in higher plants (Santesmasses et al., 2017). According to evolutionary analyses, selenoproteins and selenocysteine insertion sequence (SECIS) elements present in protozoans and animals evolved early, and were independently lost in higher plants and fungi through evolution (Novoselov et al., 2002). With regard to the nonsense suppressor tRNAs, only ochre and opal nonsense suppressor tRNA genes were found in the yerba mate genome, which suppress the phenotypes of ochre and opal mutations, respectively.

The length of the standard tRNA sequences ranged from 61 to 236 nucleotides, encoding 53 different anti-codons/isoacceptors in total, with tRNA^Ser^ having the highest abundance of genes and tRNA^Tyr^ having the lowest (Appendix 1—table 1). In our study, we also found that yerba mate tRNAs contain introns in 13 of the 55 tRNA^Met^, 9 of the 17 tRNA^Tyr^, 1 of the 65 tRNA^Ser^, and 1 of the 22 tRNA^Ile^ genes, providing additional demonstration that tRNA^Met^ and tRNA^Tyr^ are not the only intron containing tRNAs in the plant kingdom as it was previously believed (Michaud et al., 2011). The length of these introns varied from 6 to 163 nucleotides and all of them were found at the canonical position, one nucleotide 3′ to the anti-codon loop.

#### 1.2 Ribosomal RNAs

Analysis of ribosomal RNA (rRNA) family members showed the presence of 425 5S rRNA, 23 18S rRNA, and 23 25S rRNA genes. The large variability in the copy number of the rRNA genes has been observed and studied in plants for decades (Rogers and Bendich, 1987). It is believed that a high copy number of these genes is important to ensure increased demand of proteosynthesis during plant development, but also to stabilize the cell nucleus (Garcia et al., 2012). With regard to its genomic organization, the 18S and 25S rRNA genes were clustered together, while the 5S rRNA genes were tandemly located elsewhere in the genome (S-type arrangement). However, some 5S rRNA genes were linked with the 18S and 25S rRNA genes as well (L-type arrangement). Given this observation, we should incorporate the yerba mate genome to the list of eukaryotic genomes with an L-arrangement of ribosomal DNA.

#### 1.3 Small RNAs

Analysis of small RNA family members revealed the presence of 348 small nuclear RNA (snRNA) genes including 65 U1 snRNA, 46 U2 snRNA, 29 U4 snRNA, 33 U5 snRNA, and 146 U6 snRNA genes corresponding to the major spliceosome complex, and 19 U6atac snRNA, 7 U11 snRNA, and 1 U12 snRNA genes corresponding to the minor spliceosome complex. Furthermore, it showed the presence of 2670 small nucleolar RNA (snoRNA) genes, of which 2631 (~98.54%) were box C/D snoRNA genes and 39 (~1.46%) were box H/ACA snoRNA genes. Both groups of snoRNAs are involved in the cleavage of precursor ribosomal RNA (pre-rRNA) and determine site-specific modification in pre-rRNAs and snRNAs, though the box C/D snoRNAs are usually associated with 2′-*O*-ribose methylation, while the box H/ACA snoRNAs are normally associated with 2′-*O*-ribose pseudouridylation (Brown et al., 2003; Hari and Parthasarathy, 2019). The greater abundance of the box C/D snoRNAs in the yerba mate genome could be explained, first, by the fact that plants have higher numbers of 2′-*O*-ribose methylated nucleotides than archaea, yeast, and other higher eukaryotes, and second, by the fact that computer algorithms still find difficult to predict the relatively short conserved sequences of box H/ACA snoRNAs (Brown et al., 2003). It was remarkable the high copy number (2377) of snoRNA R71 in the yerba mate genome, which is a member of the Box C/D family. This snoRNA, which is thought to function as a 2′-*O*-ribose methylation guide for 18S rRNA, has been identified in multiple copies in most eudicot genomes (Kiss-László et al., 1996).

#### 1.4 Micro RNAs

Analysis of micro RNA (miRNA) family members revealed the presence of 226 miRNA genes belonging to 30 families. The most abundant miRNAs usually involved in growth and development were miR156, miR166, and miR159, while the most abundant miRNAs normally involved in stress responses were miR169_2, miR167_1, miR399, and miR395 (Appendix 1—table 2). Nevertheless, the functions of miRNAs slightly differ among plants. Therefore, to better understand the regulatory effect of miRNAs in yerba mate, we used the TAPIR web server (Bonnet et al., 2010) and the TargetFinder softwareto identify yerba mate miRNA targets (Appendix 1—table 3). The results obtained allowed us to infer the role of 14 of the 30 miRNA families found in the yerba mate genome. Apparently, miR159, miR164, miR169_2, and miR169_5 regulate a variety of processes related to development and stress responses. On the one hand, both miR159 and miR164 regulate the expression of myb-like transcription factors, involved in auxin homeostasis, lateral root and leaf development, leaf senescence, response to abscisic acid, response to the absence of light and response to salt stress. miR164 also regulates the synthesis of vitamin B5 involved in embryo development. On the other hand, both miR169_2 and miR169_5 regulate the expression of a galactinol synthase involved in the response to cold stress, heat stress, oxidative stress, salt stress and water deprivation; a kinesin-like protein involved in pollen development and a mitogen-activated protein kinase involved in directing cellular responses to mitogens, osmotic stress, heat shock, and proinflammatory cytokines. miR167_1 is probably involved only in plant growth and development as it regulates the expression of an auxin response factor (arf). And last, miR171_1 and miR390 are likely involved only in stress responses. miR171_1 regulates the expression of an RNA-binding family protein and an endoglucanase involved in host defence, whereas miR390 regulates the expression of a rotamase FKBP 1 involved in the response to heat stress, osmotic stress, and wounding. It is important to mention that the functions of all the predicted targets were gathered from the *Arabidopsis* Information Resource (TAIR) (Rhee et al., 2003), and therefore the functional involvement of these miRNAs in yerba mate must be experimentally validated.

**Appendix 1—table 1.** Detail of yerba mate tRNA and anti-codon nucleotide sequences.

tRNA genes	Anti-codon counts						Total No. of tRNAs
**POLAR**							
Asparagine (Asn)	GTT (36)	ATT (0)					36
Cysteine (Cys)	GCA (22)	ACA (0)					22
Glutamine (Gln)	TTG (13)	CTG (10)					23
Glycine (Gly)	GCC (32)	TCC (11)	CCC (8)	ACC (0)			51
Serine (Ser)	GCT (20)	TGA (20)	AGA (15)	CGA (5)	GGA (5)	ACT (0)	65
Threonine (Thr)	TGT (11)	AGT (16)	GGT (6)	CGT (2)			35
Tyrosine (Tyr)	GTA (17)	ATA (0)					17
**NON-POLAR**							
Alanine (Ala)	AGC (12)	CGC (4)	TGC (11)	GGC (0)			27
Isoleucine (Ile)	AAT (14)	TAT (6)	GAT (2)				22
Leucine (Leu)	CAA (23)	AAG (10)	CAG (4)	TAG (8)	TAA (6)	GAG (0)	51
Methionine (Met)	CAT (55)						55
Phenylalanine (Phe)	GAA (30)	AAA (2)					32
Proline (Pro)	AGG (10)	TGG (28)	CGG (4)	GGG (0)			42
Tryptophan (Trp)	CCA (31)						31
Valine (Val)	AAC (11)	GAC (10)	CAC (9)	TAC (7)			37
**POSITIVELY CHARGED**							
Arginine (Arg)	ACG (15)	TCT (14)	CCT (7)	CCG (6)	TCG (6)	GCG (3)	51
Histidine (His)	GTG (25)	ATG (2)					27
Lysine (Lys)	CTT (10)	TTT (17)					27
**NEGATIVELY CHARGED**							
Aspartic acid (Asp)	GTC (39)	ATC (1)					40
Glutamic acid (Glu)	CTC (14)	TTC (21)					35
Selenocysteine tRNAs	TCA (0)						0
Possible suppressor tRNAs	CTA (0)	TTA (1)	TCA (1)				2
tRNAs with undetermined isotypes							11
Predicted pseudogenes							76

**Appendix 1—table 2.** miRNA families predicted in the yerba mate genome.

miRNA	Functional involvement in other eudicot plants
miR156	Seed growth and development Chi et al., 2011; Song et al., 2011
	Fruit development (Pantaleo et al., 2010)
	Drought/cold stress (Curaba et al., 2012; Zhu and Luo, 2013)
miR159	Growth and development (Varkonyi-Gasic et al., 2010)
	Phase change from vegetative to reproductive growth (Han et al., 2014)
	Lipid and protein accumulation (Zhao et al., 2010)
	Drought stress (Barrera-Figueroa et al., 2011)
miR160	Growth and development (Gu et al., 2013; Wang et al., 2011)
	Fibrous root and storage root development (Sun et al., 2015)
	Drought stress (Nadarajah and Kumar, 2019)
miR162_2	Storage root initiation and development (Sun et al., 2015)
miR164	Lateral root and leaf development (Deng et al., 2015)
	Fibrous root and storage root development (Sun et al., 2015)
	Seed development (Song et al., 2011)
	Drought stress (Ferreira et al., 2012)
miR166	Seed development (Song et al., 2011)
	Fibrous root and storage root development (Sun et al., 2015)
	Drought stress (Barrera-Figueroa et al., 2011)
	Disease resistance (Guo et al., 2011)
miR167_1	Growth and development (Varkonyi-Gasic et al., 2010)
	Drought/cold stress (Barrera-Figueroa et al., 2011; Jeong et al., 2011)
miR168	Development (Gu et al., 2013)
	Resistance to fire blight (Kaja et al., 2015)
miR169_2; miR169_5	Drought/cold/salt stress (Carnavale Bottino et al., 2013; Koc et al., 2015; Sheng et al., 2015; Shui et al., 2013)
miR171_1; miR171_2	Development (Chaves et al., 2015; Zhang et al., 2011)
	Lipid and protein accumulation (Zhao et al., 2010)
miR172	Development (Sun et al., 2012)
	Starch biosynthesis (Chen et al., 2015)
	Drought/cold stress (Koc et al., 2015)
miR390	Drought stress (Shui et al., 2013)
	Leaf morphology (Karlova et al., 2013)
miR394	Drought/salt stress (Song et al., 2013)
miR395	Low sulfate response (Katiyar et al., 2012)
miR396	Seed development (Gao et al., 2015)
	Starch biosynthesis (Chen et al., 2015)
	Drought/salt stress (Shui et al., 2013; Xie et al., 2014)
miR397	Drought/cold stress (Koc et al., 2015)
miR398	Fibrous root and storage root development (Sun et al., 2015)
	Salt stress (Carnavale Bottino et al., 2013)
miR399	Phosphate homeostasis (Katiyar et al., 2012; Pant et al., 2008)
	Shoot to root transport (Pant et al., 2008)
miR403	Drought stress (Shui et al., 2013)
miR405	Transposon derived (Xie et al., 2005)
miR408	Tolerance to Boron deficiency (Lu et al., 2015)
	Cold stress (Zhang et al., 2014)
	Response to wounding and topping (Tang et al., 2012)
miR473	Metabolism (Din et al., 2014)
	Stress response (Patanun et al., 2013)
miR474	Drought stress (Kantar et al., 2011)
miR475	Metabolism (Din et al., 2014)
miR477	Starch biosynthesis (Xie et al., 2011)
miR530	Disease resistance (Zhao et al., 2015)
miR1023	Disease resistance (Jiao and Peng, 2018)
miR1446	Stress response (Lu et al., 2008)

**Appendix 1—table 3.** miRNA targets predicted in the yerba mate genome.

Targets IDs	Description	miR159	miR164	miR167_1	miR168	miR169_2	miR169_5	miR171_1	miR171_2	miR390	miR394	miR396	miR397	miR398	miR403
ILEXPARA_008283	Uncharacterized protein	✸													
ILEXPARA_029002	Uncharacterized protein	✸													
ILEXPARA_031381	Uncharacterized protein	✸													
ILEXPARA_043376	Uncharacterized protein	✸													
ILEXPARA_013180	*ileS*, isoleucine tRNA ligase	✸													
ILEXPARA_000910	myb-like transcription factor	✸	✸												
ILEXPARA_028644	Uncharacterized protein		✸												
ILEXPARA_005969	Putative membrane protein		✸												
ILEXPARA_048009	*panC*, pantothenate (vitamin B5) synthetase		✸												
ILEXPARA_018064	*arf*, auxin response factor			✸											
ILEXPARA_019275	Uncharacterized protein			✸											
ILEXPARA_024153	Uncharacterized protein			✸											
ILEXPARA_035190	Uncharacterized protein			✸											
ILEXPARA_016483	Hypothetical protein				✸										
ILEXPARA_029421	*GCP4*, gamma tubulin complex protein 4					✸	✸								
ILEXPARA_047849	*GOLS1*, galactinol synthase 1					✸	✸								
ILEXPARA_003987	*NACK1*, kinesin-like protein					✸	✸								
ILEXPARA_044341	Uncharacterized protein					✸									
ILEXPARA_005359	Uncharacterized protein					✸									
ILEXPARA_035716	*RABE1C*, ras-related protein					✸									
ILEXPARA_010316	*MAPK*, mitogen activated protein kinase					✸	✸								
ILEXPARA_032923	Uncharacterized protein					✸	✸								
ILEXPARA_008149	Uncharacterized protein					✸	✸								
ILEXPARA_048631	Protein kinase					✸	✸								
ILEXPARA_008152	Uncharacterized protein							✸							
ILEXPARA_023090	*NAGK*, *N*-acetyl-D-glucosamine kinase							✸							
ILEXPARA_024088	RNA-binding (RRM/RBD/RNP motif) family protein							✸							
ILEXPARA_023716	Endoglucanase							✸							
ILEXPARA_042182	Uncharacterized protein							✸							
ILEXPARA_021515	Pentatricopeptide repeat (PPR) protein								✸						
ILEXPARA_004925	Uncharacterized protein									✸					
ILEXPARA_045111	Rotamase *FKBP 1*									✸					
ILEXPARA_013832	*ABCC2*, ABC transporter C family member 2 protein										✸				
ILEXPARA_039828	*guaA*, GMP synthase										✸				
ILEXPARA_028274	Hypothetical protein										✸				
ILEXPARA_024538	*RPT6A*, regulatory particle triple-A ATPase 6A											✸			
ILEXPARA_031387	Uncharacterized protein													✸	
ILEXPARA_043757	Uncharacterized protein													✸	
ILEXPARA_005297	Uncharacterized protein													✸	
ILEXPARA_012032	Uncharacterized protein													✸	
ILEXPARA_9682	*OST1B*, oligosaccharyltransferase 1B														✸

**Appendix 1—key resources table:**

Reagent type (species) or resource	Designation	Source or reference	Identifiers	Additional information
Gene (*Ilex paraguariensis*)	IpCS1	GenBank	CAK9135737	Xanthine methyltransferase gene of *Ilex paraguariensis*
Gene (*Ilex paraguariensis*)	IpCS2	GenBank	CAK9135740	3-Methylxanthine methyltransferase gene of *Ilex paraguariensis*
Gene (*Ilex paraguariensis*)	IpCS3	GenBank	CAK9135742	Theobromine methyltransferase gene of *Ilex paraguariensis*
Strain, strain background (*Escherichia coli*)	BL21(DE3)	Novagen	69450-M	Chemically competent cells
Biological sample (*Ilex paraguariensis*)	*Ilex paraguariensis* A. St.-Hil. var. *paraguariensis*	INTA-EEA Cerro Azul, Misiones, Argentina	cv CA 8/74	Used to extract genomic DNA
Biological sample (*Ilex paraguariensis*)	*Ilex paraguariensis* A. St.-Hil. var. *paraguariensis*	Establecimiento Las Marías S.A.C.I.F.A., Corrientes, Argentina	cv SI-49	Used to extract genomic DNA
Recombinant DNA reagent	pUC57-IpCS1 (plasmid)	GenScript		Used to clone IpCS1 gene
Recombinant DNA reagent	pTrcHis-IpCS2 (plasmid)	This paper		Used to clone IpCS2 gene
Recombinant DNA reagent	pUC57-IpCS3 (plasmid)	GenScript		Used to clone IpCS3 gene
Recombinant DNA reagent	pUC57-AncIpCS1 (plasmid)	GenScript		Used to clone AncIpCS1 gene
Recombinant DNA reagent	pUC57-AncIpCS2 (plasmid)	GenScript		Used to clone AncIpCS2 gene
Sequence-based reagent	pET-15b- IpCS1 (plasmid)	This paper		Used to express IpCS1 in *E. coli* BL21(DE3)
Sequence-based reagent	pET-15b- IpCS2 (plasmid)	This paper		Used to express IpCS2 in *E. coli* BL21(DE3)
Sequence-based reagent	pET-15b- IpCS3 (plasmid)	This paper		Used to express IpCS3 in *E. coli* BL21(DE3)
Sequence-based reagent	pET-15b- AncIpCS1 (plasmid)	This paper		Used to express AncIpCS1 in *E. coli* BL21(DE3)
Sequence-based reagent	pET-15b- AncIpCS2 (plasmid)	This paper		Used to express AncIpCS2 in *E. coli* BL21(DE3)
Sequence-based reagent	IpCS2F	This paper	PCR primers	5′-ATGGACGTGAAGGAAGCAC-3′
Sequence-based reagent	IpCS2R	This paper	PCR primers	5′-CTATCCCATGGTCCTGCTAAG-3′
Peptide, recombinant protein	IpCS1	This paper		Purified from *E. coli* BL21(DE3) cells
Peptide, recombinant protein	IpCS2	This paper		Purified from *E. coli* BL21(DE3) cells
Peptide, recombinant protein	IpCS3	This paper		Purified from *E. coli* BL21(DE3) cells
Peptide, recombinant protein	AncIpCS1	This paper		Purified from *E. coli* BL21(DE3) cells
Peptide, recombinant protein	AncIpCS2	This paper		Purified from *E. coli* BL21(DE3) cells
Commercial assay or kit	DNeasy Plant Mini Kit	QIAGEN	Cat. #: 69104	Used to extract genomic DNA from *Ilex paraguariensis*
Commercial assay or kit	Quick-DNA HMW MagBead Kit	Zymo Research	Cat. #: D6060	Used to extract genomic DNA from *Ilex paraguariensis*
Commercial assay or kit	Illumina TruSeq DNA Sample Preparation Kit	Illumina	Cat. #: FC-121-2003	Used to construct paired-end libraries
Commercial assay or kit	Illumina Nextera Mate Pair Library Preparation Kit	Illumina	Cat. #: FC-132-1001	Used to construct mate-pair libraries
Commercial assay or kit	Sequel Binding Kit 1.0	Pacific Biosciences	Cat. #: 101-365-900	Used for preparing DNA templates for sequencing on the PacBio Sequel System
Commercial assay or kit	Sequel Sequencing Kit 1.0	Pacific Biosciences	Cat. #: 101-309-500	Used to perform sequencing reactions on the PacBio Sequel System
Commercial assay or kit	SMRT Cell 1M	Pacific Biosciences	Cat. #: 100-171-800	Consumable microchip used in the PacBio Sequel System for Single Molecule, Real-Time (SMRT) sequencing
Commercial assay or kit	pTrcHis TOPO TA Expression Kit	Invitrogen	Cat. #: K4410-01	Used to clone IpCS2 gene
Commercial assay or kit	QIAEX II Gel Extraction Kit	QIAGEN	Cat. #: 20021	Used to clone IpCS1, IpCS3, AncIpCS1, and AncIpCS2 genes into pET-15b expression vector
Commercial assay or kit	Agilent QuikChange Lightning Kit	Agilent Technologies Inc, Santa Clara, CA	Cat. #: 210518	Used for site-directed mutagenesis of AncIpCS2
Commercial assay or kit	QIAprep Spin Miniprep Kit	QIAGEN	Cat. #: 27104	Used for the rapid purification of high-quality plasmid DNA
Commercial assay or kit	TALON spin columns	Takara Bio	Cat. #: 89068	Used for the purification of histidine-tagged proteins
Chemical compound, drug	Xanthine	Sigma-Aldrich	Cat. #: X0626	Used to test relative substrate preference of IpCS1–3 and AncIpCS1–2
Chemical compound, drug	Xanthosine	Sigma-Aldrich	Cat. #: X0750	Used to test relative substrate preference of IpCS1–3 and AncIpCS1–2
Chemical compound, drug	1-Methylxanthine	Sigma-Aldrich	Cat. #: 69720	Used to test relative substrate preference of IpCS1–3 and AncIpCS1–2
Chemical compound, drug	3-Methylxanthine	Sigma-Aldrich	Cat. #: 222526	Used to test relative substrate preference of IpCS1–3 and AncIpCS1–2
chemical compound, drug	7-Methylxanthine	Sigma-Aldrich	Cat. #: 69723	Used to test relative substrate preference of IpCS1–3 and AncIpCS1–2
Chemical compound, drug	Theobromine	Sigma-Aldrich	Cat. #: T4500	Used to test relative substrate preference of IpCS1–3 and AncIpCS1–2
Chemical compound, drug	Paraxanthine	Sigma-Aldrich	Cat. #: D5385	Used to test relative substrate preference of IpCS1–3 and AncIpCS1–2
Chemical compound, drug	Theophylline	Sigma-Aldrich	Cat. #: T1633	Used to test relative substrate preference of IpCS1–3 and AncIpCS1–2
Software, algorithm	Trimmomatic	DOI: 10.1093/bioinformatics/btu170	v.0.39	Used to remove adaptor contaminations and filter low-quality reads
Software, algorithm	Quake	DOI: 10.1186/gb-2010-11-11-r116	v.0.3	Used to correct clean reads
Software, algorithm	SOAPdenovo	DOI: 10.1186/2047-217X-1-18	v.2	Used to assemble and scaffold contigs
Software, algorithm	DeconSeq	DOI: 10.1371/journal.pone.0017288	v.0.4.3	Used to detect and remove sequence contaminants
Software, algorithm	Canu	DOI: 10.1101/gr.215087.116	v.2.2	Used for self-correction and assembly of long reads
Software, algorithm	PurgeHaplotigs	DOI: 10.1186/s12859-018-2441-2		Used to separate assembly haplotypes
Software, algorithm	Quickmerge	DOI: 10.1101/029306	v.03	Used to merge SOAPdenovo and Canu curated assemblies
Software, algorithm	SSPACE	DOI: 10.1093/bioinformatics/btq683	v.2.1.1	Used to refine scaffolds and contigs
Software, algorithm	RepeatMasker	http://repeatmasker.org/		Used to mask the genome assembly
Software, algorithm	Funannotate	DOI: 10.5281/zenodo.2604804	v.1.8.13	Used to predict the protein- and non-coding genes
Software, algorithm	Infernal	DOI: 10.1093/bioinformatics/btt509	v.1.1.4	Used to improve the prediction of small RNAs and microRNAs
Software, algorithm	tRNAScan-SE	DOI: 10.1007/978-1-4939-9173-0_1	v.2.0	Used to improve the prediction of transfer RNAs
software, algorithm	TAPIR	http://bioinformatics.psb.ugent.be/webtools/tapir		Used to identify miRNA targets
Software, algorithm	TargetFinder	DOI: 10.1007/978-1-60327-005-2_4	v.1.7	Used to identify miRNA targets
Software, algorithm	InterProScan	DOI: 10.1093/bioinformatics/btu031	v.5.55-88.0	Used to assign function of the predicted genes
Software, algorithm	eggNOG-mapper	DOI: 10.1093/nar/gky1085	v.2.1.7	Used to assign function to the predicted genes
Software, algorithm	Dfam TE Tools	https://github.com/Dfam-consortium/TETools	v.1.5	Used to estimate the repeat content
Software, algorithm	CoGe’s tool SynMap	https://genomevolution.org/		Used to estimate rates of synonymous substitution (Ks) between paralogous and orthologous genes
Software, algorithm	CoGe's tool SynFind	https://genomevolution.org/		Used to determine the syntenic depth ratio between *I. paraguariensis*, *C. canephora*, and *V. vinifera*
Software, algorithm	CoGe’s tool GEvo	https://genomevolution.org/		Used to compare CS and XMT syntenic regions
Software, algorithm	MAFFT	DOI: 10.1093/molbev/mst010	v.7.0	Used to align amino acid sequences
Software, algorithm	FastTree	DOI: 10.1371/journal.pone.0009490	v.2	Used to perform phylogenetic analysis of SABATH sequences
Software, algorithm	IQTree	DOI: 10.1093/nar/gkw256		Used to estimate ancestral sequences
Software, algorithm	Phenix	DOI: 10.1107/S0907444909052925		Used to solve the crystal structure of IpCS3
software, algorithm	REFMAC5	DOI: 10.1107/S0907444911001314		Used to refine the crystal structure of IpCS3
Software, algorithm	COOT	DOI: 10.1107/S0907444910007493	v.0.9.8.3	Used to refine the crystal structure of IpCS3
Other	*Ilex paraguariensis* transcriptome sequence data	ENA	PRJNA315513	Used to assess the completeness of *Ilex paraguariensis* genome
Other	*Ilex paraguariensis* transcriptome sequence data	NCBI	SRP043293	Used to assess the completeness of *Ilex paraguariensis* genome
Other	*Ilex paraguariensis* transcriptome sequence data	NCBI	SRP110129	Used to determine the expression of IpCS1–5 genes
Other	Vivaspin columns	Sartorius	Cat. #: VS0101	Used to remove proteins after enzymatic reaction
Other	Kinetex 5 μM EVO C18 column	Phenomenex	Cat. #: 00F-4467-AN	Used for high-performance liquid chromatography
Other	Crystal Gryphon robot	Art Robbins Instruments	Cat. #: 100-1010	Used for automating crystallization

## References

[bib1] Acevedo RM, Avico EH, González S, Salvador AR, Rivarola M, Paniego N, Nunes-Nesi A, Ruiz OA, Sansberro PA (2019). Transcript and metabolic adjustments triggered by drought in Ilex paraguariensis leaves. Planta.

[bib2] Adams PD, Afonine PV, Bunkóczi G, Chen VB, Davis IW, Echols N, Headd JJ, Hung LW, Kapral GJ, Grosse-Kunstleve RW, McCoy AJ, Moriarty NW, Oeffner R, Read RJ, Richardson DC, Richardson JS, Terwilliger TC, Zwart PH (2010). PHENIX: a comprehensive python-based system for macromolecular structure solution. Acta Crystallographica. Section D, Biological Crystallography.

[bib3] Altschul SF, Gish W, Miller W, Myers EW, Lipman DJ (1990). Basic local alignment search tool. Journal of Molecular Biology.

[bib4] Anaya AL, Cruz-Ortega R, Waller GR (2006). Metabolism and ecology of purine alkaloids. Frontiers in Bioscience.

[bib5] Ashihara H, Monteiro AM, Gillies FM, Crozier A (1996). Biosynthesis of caffeine in leaves of coffee. Plant Physiology.

[bib6] Avico EH, Acevedo RM, Duarte MJ, Rodrigues Salvador A, Nunes-Nesi A, Ruiz OA, Sansberro PA (2023). Integrating transcriptional, metabolic, and physiological responses to drought stress in *Ilex paraguariensis* roots. Plants.

[bib7] Barrera-Figueroa BE, Gao L, Diop NN, Wu Z, Ehlers JD, Roberts PA, Close TJ, Zhu JK, Liu R (2011). Identification and comparative analysis of drought-associated microRNAs in two cowpea genotypes. BMC Plant Biology.

[bib8] Boetzer M, Henkel CV, Jansen HJ, Butler D, Pirovano W (2011). Scaffolding pre-assembled contigs using SSPACE. Bioinformatics.

[bib9] Bolger AM, Lohse M, Usadel B (2014). Trimmomatic: a flexible trimmer for Illumina sequence data. Bioinformatics.

[bib10] Bonnet E, He Y, Billiau K, Van de Peer Y (2010). TAPIR, a web server for the prediction of plant microRNA targets, including target mimics. Bioinformatics.

[bib11] Brown JWS, Echeverria M, Qu LH, Lowe TM, Bachellerie JP, Hüttenhofer A, Kastenmayer JP, Green PJ, Shaw P, Marshall DF (2003). Plant snoRNA database. Nucleic Acids Research.

[bib12] Buerki S, Forest F, Alvarez N, Nylander JAA, Arrigo N, Sanmartín I (2011). An evaluation of new parsimony-based versus parametric inference methods in biogeography: a case study using the globally distributed plant family Sapindaceae. Journal of Biogeography.

[bib13] Carnavale Bottino M, Rosario S, Grativol C, Thiebaut F, Rojas CA, Farrineli L, Hemerly AS, Ferreira PCG (2013). High-throughput sequencing of small RNA transcriptome reveals salt stress regulated microRNAs in sugarcane. PLOS ONE.

[bib14] Chakraborty M, Baldwin-Brown JG, Long AD, Emerson JJ (2016). Contiguous and accurate de novo assembly of metazoan genomes with modest long read coverage. Genomics.

[bib15] Chan PP, Lowe TM (2019). tRNAscan-SE: Searching for tRNA Genes in Genomic Sequences. Methods in Molecular Biology.

[bib16] Chaudhuri P, Marron JS (2000). Scale space view of curve estimation. The Annals of Statistics.

[bib17] Chaves SS, Fernandes-Brum CN, Silva GFF, Ferrara-Barbosa BC, Paiva LV, Nogueira FTS, Cardoso TCS, Amaral LR, de Souza Gomes M, Chalfun-Junior A (2015). New insights on coffea mirnas: features and evolutionary conservation. Applied Biochemistry and Biotechnology.

[bib18] Chen X, Xia J, Xia Z, Zhang H, Zeng C, Lu C, Zhang W, Wang W (2015). Potential functions of microRNAs in starch metabolism and development revealed by miRNA transcriptome profiling of cassava cultivars and their wild progenitor. BMC Plant Biology.

[bib19] Chi X, Yang Q, Chen X, Wang J, Pan L, Chen M, Yang Z, He Y, Liang X, Yu S (2011). Identification and characterization of microRNAs from peanut (Arachis hypogaea L.) by high-throughput sequencing. PLOS ONE.

[bib20] Curaba J, Spriggs A, Taylor J, Li Z, Helliwell C (2012). miRNA regulation in the early development of barley seed. BMC Plant Biology.

[bib21] Daly JW, Butts-Lamb P, Padgett W (1983). Subclasses of adenosine receptors in the central nervous system: interaction with caffeine and related methylxanthines. Cellular and Molecular Neurobiology.

[bib22] Dean AM, Thornton JW (2007). Mechanistic approaches to the study of evolution: the functional synthesis. Nature Reviews. Genetics.

[bib23] Debat HJ, Grabiele M, Aguilera PM, Bubillo RE, Otegui MB, Ducasse DA, Zapata PD, Marti DA (2014). Exploring the genes of yerba mate (Ilex paraguariensis A. St.-Hil.) by NGS and de novo transcriptome assembly. PLOS ONE.

[bib24] Deng P, Wang L, Cui L, Feng K, Liu F, Du X, Tong W, Nie X, Ji W, Weining S (2015). Global identification of microRNAs and their targets in barley under salinity stress. PLOS ONE.

[bib25] Din M, Barozai MYK, Baloch IA (2014). Identification and functional analysis of new conserved microRNAs and their targets in potato (Solanum tuberosum L.). TURKISH JOURNAL OF BOTANY.

[bib26] Dubs NM, Davis BR, de Brito V, Colebrook KC, Tiefel IJ, Nakayama MB, Huang R, Ledvina AE, Hack SJ, Inkelaar B, Martins TR, Aartila SM, Albritton KS, Almuhanna S, Arnoldi RJ, Austin CK, Battle AC, Begeman GR, Bickings CM, Bradfield JT, Branch EC, Conti EP, Cooley B, Dotson NM, Evans CJ, Fries AS, Gilbert IG, Hillier WD, Huang P, Hyde KW, Jevtovic F, Johnson MC, Keeler JL, Lam A, Leach KM, Livsey JD, Lo JT, Loney KR, Martin NW, Mazahem AS, Mokris AN, Nichols DM, Ojha R, Okorafor NN, Paris JR, Reboucas TF, Sant’Anna PB, Seitz MR, Seymour NR, Slaski LK, Stemaly SO, Ulrich BR, Van Meter EN, Young ML, Barkman TJ (2022). A collaborative classroom investigation of the evolution of sabath methyltransferase substrate preference shifts over 120 my of flowering plant history. Molecular Biology and Evolution.

[bib27] Eberhardt J, Santos-Martins D, Tillack AF, Forli S (2021). AutoDock vina 1.2.0: new docking methods, expanded force field, and python bindings. Journal of Chemical Information and Modeling.

[bib28] Emsley P, Lohkamp B, Scott WG, Cowtan K (2010). Features and development of Coot. Acta Crystallographica. Section D, Biological Crystallography.

[bib29] Fahlgren N, Carrington JC (2010). miRNA target prediction in plants. Methods in Molecular Biology.

[bib30] Fay JV, Watkins CJ, Shrestha RK, Litwiñiuk SL, Talavera Stefani LN, Rojas CA, Argüelles CF, Ferreras JA, Caccamo M, Miretti MM (2018). Yerba mate (Ilex paraguariensis, A. St.-Hil.) de novo transcriptome assembly based on tissue specific genomic expression profiles. BMC Genomics.

[bib31] Ferreira TH, Gentile A, Vilela RD, Costa GGL, Dias LI, Endres L, Menossi M (2012). microRNAs associated with drought response in the bioenergy crop sugarcane (Saccharum spp.). PLOS ONE.

[bib32] Flynn JM, Hubley R, Goubert C, Rosen J, Clark AG, Feschotte C, Smit AF (2020). RepeatModeler2 for automated genomic discovery of transposable element families. PNAS.

[bib33] Galindo-González L, Mhiri C, Deyholos MK, Grandbastien MA (2017). LTR-retrotransposons in plants: engines of evolution. Gene.

[bib34] Gao F, Wang K, Liu Y, Chen Y, Chen P, Shi Z, Luo J, Jiang D, Fan F, Zhu Y, Li S (2015). Blocking miR396 increases rice yield by shaping inflorescence architecture. Nature Plants.

[bib35] Garcia S, Crhák Khaitová L, Kovařík A (2012). Expression of 5 S rRNA genes linked to 35 S rDNA in plants, their epigenetic modification and regulatory element divergence. BMC Plant Biology.

[bib36] Goodstein DM, Shu S, Howson R, Neupane R, Hayes RD, Fazo J, Mitros T, Dirks W, Hellsten U, Putnam N, Rokhsar DS (2012). Phytozome: a comparative platform for green plant genomics. Nucleic Acids Research.

[bib37] Gottlieb AM, Poggio L (2015). Quantitative and qualitative genomic characterization of cultivated *Ilex* L. species. Plant Genetic Resources.

[bib38] Gu Y, Liu Y, Zhang J, Liu H, Hu Y, Du H, Li Y, Chen J, Wei B, Huang Y (2013). Identification and characterization of microRNAs in the developing maize endosperm. Genomics.

[bib39] Gugliucci A (1996). Antioxidant effects of Ilex paraguariensis: induction of decreased oxidability of human LDL in vivo. Biochemical and Biophysical Research Communications.

[bib40] Guo H, Kan Y, Liu W (2011). Differential expression of miRNAs in response to topping in flue-cured tobacco (Nicotiana tabacum) roots. PLOS ONE.

[bib41] Hamon P, Grover CE, Davis AP, Rakotomalala JJ, Raharimalala NE, Albert VA, Sreenath HL, Stoffelen P, Mitchell SE, Couturon E, Hamon S, de Kochko A, Crouzillat D, Rigoreau M, Sumirat U, Akaffou S, Guyot R (2017). Genotyping-by-sequencing provides the first well-resolved phylogeny for coffee (Coffea) and insights into the evolution of caffeine content in its species: GBS coffee phylogeny and the evolution of caffeine content. Molecular Phylogenetics and Evolution.

[bib42] Han J, Fang J, Wang C, Yin Y, Sun X, Leng X, Song C (2014). Grapevine microRNAs responsive to exogenous gibberellin. BMC Genomics.

[bib43] Hari R, Parthasarathy S (2019). Prediction of coding and non-coding RNA. Encyclopedia of Bioinformatics and Computational Biology.

[bib44] Heck CI, de Mejia EG (2007). Yerba Mate Tea (Ilex paraguariensis): a comprehensive review on chemistry, health implications, and technological considerations. Journal of Food Science.

[bib45] Huang R, O’Donnell AJ, Barboline JJ, Barkman TJ (2016). Convergent evolution of caffeine in plants by co-option of exapted ancestral enzymes. PNAS.

[bib46] Hubley R, Finn RD, Clements J, Eddy SR, Jones TA, Bao W, Smit AFA, Wheeler TJ (2016). The Dfam database of repetitive DNA families. Nucleic Acids Research.

[bib47] Huerta-Cepas J, Szklarczyk D, Heller D, Hernández-Plaza A, Forslund SK, Cook H, Mende DR, Letunic I, Rattei T, Jensen LJ, von Mering C, Bork P (2019). eggNOG 5.0: a hierarchical, functionally and phylogenetically annotated orthology resource based on 5090 organisms and 2502 viruses. Nucleic Acids Research.

[bib48] Humphrey W, Dalke A, Schulten K (1996). VMD: visual molecular dynamics. Journal of Molecular Graphics.

[bib49] Iorizzo M, Ellison S, Senalik D, Zeng P, Satapoomin P, Huang J, Bowman M, Iovene M, Sanseverino W, Cavagnaro P, Yildiz M, Macko-Podgórni A, Moranska E, Grzebelus E, Grzebelus D, Ashrafi H, Zheng Z, Cheng S, Spooner D, Van Deynze A, Simon P (2016). A high-quality carrot genome assembly provides new insights into carotenoid accumulation and asterid genome evolution. Nature Genetics.

[bib50] Jeong DH, Park S, Zhai J, Gurazada SGR, De Paoli E, Meyers BC, Green PJ (2011). Massive analysis of rice small RNAs: mechanistic implications of regulated microRNAs and variants for differential target RNA cleavage. The Plant Cell.

[bib51] Jiao J, Peng D (2018). Wheat microRNA1023 suppresses invasion of Fusarium graminearum via targeting and silencing FGSG_03101. J Plant Interact.

[bib52] Jin JQ, Yao MZ, Ma CL, Ma JQ, Chen L (2016). Natural allelic variations of TCS1 play a crucial role in caffeine biosynthesis of tea plant and its related species. Plant Physiology and Biochemistry.

[bib53] Jones P, Binns D, Chang HY, Fraser M, Li W, McAnulla C, McWilliam H, Maslen J, Mitchell A, Nuka G, Pesseat S, Quinn AF, Sangrador-Vegas A, Scheremetjew M, Yong SY, Lopez R, Hunter S (2014). InterProScan 5: genome-scale protein function classification. Bioinformatics.

[bib54] Jurka J, Kapitonov VV, Pavlicek A, Klonowski P, Kohany O, Walichiewicz J (2005). Repbase Update, a database of eukaryotic repetitive elements. Cytogenetic and Genome Research.

[bib55] Kaja E, Szcześniak MW, Jensen PJ, Axtell MJ, McNellis T, Makałowska I (2015). Identification of apple miRNAs and their potential role in fire blight resistance. Tree Genetics & Genomes.

[bib56] Kang YR, Lee HY, Kim JH, Moon DI, Seo MY, Park SH, Choi KH, Kim CR, Kim SH, Oh JH, Cho SW, Kim SY, Kim MG, Chae SW, Kim O, Oh HG (2012). Anti-obesity and anti-diabetic effects of Yerba Mate (Ilex paraguariensis) in C57BL/6J mice fed a high-fat diet. Laboratory Animal Research.

[bib57] Kantar M, Lucas SJ, Budak H (2011). miRNA expression patterns of Triticum dicoccoides in response to shock drought stress. Planta.

[bib58] Karlova R, van Haarst JC, Maliepaard C, van de Geest H, Bovy AG, Lammers M, Angenent GC, de Maagd RA (2013). Identification of microRNA targets in tomato fruit development using high-throughput sequencing and degradome analysis. Journal of Experimental Botany.

[bib59] Katiyar A, Smita S, Chinnusamy V, Pandey DM, Bansal K (2012). Identification of miRNAs in sorghum by using bioinformatics approach. Plant Signaling & Behavior.

[bib60] Kato M, Kanehara T, Shimizu H, Suzuki T, Gillies FM, Crozier A, Ashihara H (1996). Caffeine biosynthesis in young leaves of Camellia sinensis: In vitro studies on N-methyltransferase activity involved in the conversion of xanthosine to caffeine. Physiologia Plantarum.

[bib61] Katoh K, Standley DM (2013). MAFFT multiple sequence alignment software version 7: improvements in performance and usability. Molecular Biology and Evolution.

[bib62] Kelley DR, Schatz MC, Salzberg SL (2010). Quake: quality-aware detection and correction of sequencing errors. Genome Biology.

[bib63] Kim YS, Uefuji H, Ogita S, Sano H (2006). Transgenic tobacco plants producing caffeine: a potential new strategy for insect pest control. Transgenic Research.

[bib64] Kim YS, Lim S, Kang KK, Jung YJ, Lee YH, Choi YE, Sano H (2011). Resistance against beet armyworms and cotton aphids in caffeine-producing transgenic chrysanthemum. Plant Biotechnology.

[bib65] Kiss-László Z, Henry Y, Bachellerie JP, Caizergues-Ferrer M, Kiss T (1996). Site-specific ribose methylation of preribosomal RNA: a novel function for small nucleolar RNAs. Cell.

[bib66] Koc I, Filiz E, Tombuloglu H (2015). Assessment of miRNA expression profile and differential expression pattern of target genes in cold-tolerant and cold-sensitive tomato cultivars. Biotechnology & Biotechnological Equipment.

[bib67] Kong BLH, Nong W, Wong KH, Law STS, So WL, Chan JJS, Zhang J, Lau TWD, Hui JHL, Shaw PC (2022). Chromosomal level genome of Ilex asprella and insight into antiviral triterpenoid pathway. Genomics.

[bib68] Koren S, Walenz BP, Berlin K, Miller JR, Bergman NH, Phillippy AM (2017). Canu: scalable and accurate long-read assembly via adaptive *k*-mer weighting and repeat separation. Genome Research.

[bib69] Lagesen K, Hallin P, Rødland EA, Staerfeldt HH, Rognes T, Ussery DW (2007). RNAmmer: consistent and rapid annotation of ribosomal RNA genes. Nucleic Acids Research.

[bib70] Landis JB, Soltis DE, Li Z, Marx HE, Barker MS, Tank DC, Soltis PS (2018). Impact of whole-genome duplication events on diversification rates in angiosperms. American Journal of Botany.

[bib71] Lanzarotti E, Defelipe LA, Marti MA, Turjanski AG (2020). Aromatic clusters in protein-protein and protein-drug complexes. Journal of Cheminformatics.

[bib72] Li H, Durbin R (2009). Fast and accurate short read alignment with Burrows-Wheeler transform. Bioinformatics.

[bib73] Lu S, Sun YH, Chiang VL (2008). Stress-responsive microRNAs in Populus. The Plant Journal.

[bib74] Lu YB, Qi YP, Yang LT, Guo P, Li Y, Chen LS (2015). Boron-deficiency-responsive microRNAs and their targets in Citrus sinensis leaves. BMC Plant Biology.

[bib75] Luo R, Liu B, Xie Y, Li Z, Huang W, Yuan J, He G, Chen Y, Pan Q, Liu Y, Tang J, Wu G, Zhang H, Shi Y, Liu Y, Yu C, Wang B, Lu Y, Han C, Cheung DW, Yiu SM, Peng S, Xiaoqian Z, Liu G, Liao X, Li Y, Yang H, Wang J, Lam TW, Wang J (2012). SOAPdenovo2: an empirically improved memory-efficient short-read de novo assembler. GigaScience.

[bib76] Luo H, Lin Y, Gao F, Zhang CT, Zhang R (2014). DEG 10, an update of the database of essential genes that includes both protein-coding genes and noncoding genomic elements. Nucleic Acids Research.

[bib77] Magallón S, Gómez-Acevedo S, Sánchez-Reyes LL, Hernández-Hernández T (2015). A metacalibrated time-tree documents the early rise of flowering plant phylogenetic diversity. The New Phytologist.

[bib78] Manni M, Berkeley MR, Seppey M, Simão FA, Zdobnov EM (2021). BUSCO update: novel and streamlined workflows along with broader and deeper phylogenetic coverage for scoring of eukaryotic, prokaryotic, and viral genomes. Molecular Biology and Evolution.

[bib79] McCarthy AA, McCarthy JG (2007). The structure of two N-methyltransferases from the caffeine biosynthetic pathway. Plant Physiology.

[bib80] Mendez D, Gaulton A, Bento AP, Chambers J, De Veij M, Félix E, Magariños MP, Mosquera JF, Mutowo P, Nowotka M, Gordillo-Marañón M, Hunter F, Junco L, Mugumbate G, Rodriguez-Lopez M, Atkinson F, Bosc N, Radoux CJ, Segura-Cabrera A, Hersey A, Leach AR (2019). ChEMBL: towards direct deposition of bioassay data. Nucleic Acids Research.

[bib81] Michaud M, Cognat V, Duchêne AM, Maréchal-Drouard L (2011). A global picture of tRNA genes in plant genomes. The Plant Journal.

[bib82] Mirdita M, Schütze K, Moriwaki Y, Heo L, Ovchinnikov S, Steinegger M (2022). ColabFold: making protein folding accessible to all. Nature Methods.

[bib83] Morris GM, Huey R, Lindstrom W, Sanner MF, Belew RK, Goodsell DS, Olson AJ (2009). AutoDock4 and AutoDockTools4: Automated docking with selective receptor flexibility. Journal of Computational Chemistry.

[bib84] Murshudov GN, Skubák P, Lebedev AA, Pannu NS, Steiner RA, Nicholls RA, Winn MD, Long F, Vagin AA (2011). REFMAC5 for the refinement of macromolecular crystal structures. Acta Crystallographica. Section D, Biological Crystallography.

[bib85] Nadarajah K, Kumar IS (2019). Drought response in rice: the miRNA story. International Journal of Molecular Sciences.

[bib86] Nawrocki EP, Eddy SR (2013). Infernal 1.1: 100-fold faster rna homology searches. Bioinformatics.

[bib87] Negrin A, Long C, Motley TJ, Kennelly EJ (2019). LC-ms metabolomics and chemotaxonomy of caffeine-containing holly ( ilex) species and related taxa in the aquifoliaceae. Journal of Agricultural and Food Chemistry.

[bib88] Niemenak N, Onomo PE, Lieberei R, Ndoumou DO (2008). Purine alkaloids and phenolic compounds in three Cola species and Garcinia kola grown in Cameroon. South African Journal of Botany.

[bib89] Niklas CO (1987). Estudios embriológicos y citológicos en la yerba mate Ilex Paraguariensis (Aquifoliaceae). Bonplandia.

[bib90] Noda-Garcia L, Liebermeister W, Tawfik DS (2018). Metabolite-enzyme coevolution: from single enzymes to metabolic pathways and networks. Annual Review of Biochemistry.

[bib91] Novoselov SV, Rao M, Onoshko NV, Zhi H, Kryukov GV, Xiang Y, Weeks DP, Hatfield DL, Gladyshev VN (2002). Selenoproteins and selenocysteine insertion system in the model plant cell system, *Chlamydomonas reinhardtii*. The EMBO Journal.

[bib92] O’Donnell AJ, Huang R, Barboline JJ, Barkman TJ (2021). Convergent biochemical pathways for xanthine alkaloid production in plants evolved from ancestral enzymes with different catalytic properties. Molecular Biology and Evolution.

[bib93] One Thousand Plant Transcriptomes Initiative (2019). One thousand plant transcriptomes and the phylogenomics of green plants. Nature.

[bib94] Palmer J, Stajich J (2019). Funannotate.

[bib95] Pant BD, Buhtz A, Kehr J, Scheible WR (2008). MicroRNA399 is a long-distance signal for the regulation of plant phosphate homeostasis. The Plant Journal.

[bib96] Pantaleo V, Szittya G, Moxon S, Miozzi L, Moulton V, Dalmay T, Burgyan J (2010). Identification of grapevine microRNAs and their targets using high-throughput sequencing and degradome analysis. The Plant Journal.

[bib97] Patanun O, Lertpanyasampatha M, Sojikul P, Viboonjun U, Narangajavana J (2013). Computational identification of microRNAs and their targets in cassava (Manihot esculenta Crantz.). Molecular Biotechnology.

[bib98] Petronikolou N, Hollatz AJ, Schuler MA, Nair SK (2018). Loganic acid methyltransferase: insights into the specificity of methylation on an iridoid glycoside. Chembiochem.

[bib99] Pfeil BE, Crisp MD (2008). The age and biogeography of Citrus and the orange subfamily (Rutaceae: Aurantioideae) in Australasia and New Caledonia. American Journal of Botany.

[bib100] Pichersky E, Lewinsohn E (2011). Convergent evolution in plant specialized metabolism. Annual Review of Plant Biology.

[bib101] Price MN, Dehal PS, Arkin AP (2010). FastTree 2--approximately maximum-likelihood trees for large alignments. PLOS ONE.

[bib102] Ran JH, Shen TT, Wang MM, Wang XQ (2018). Phylogenomics resolves the deep phylogeny of seed plants and indicates partial convergent or homoplastic evolution between Gnetales and angiosperms. Proceedings. Biological Sciences.

[bib103] Reyes-Chin-Wo S, Wang Z, Yang X, Kozik A, Arikit S, Song C, Xia L, Froenicke L, Lavelle DO, Truco MJ, Xia R, Zhu S, Xu C, Xu H, Xu X, Cox K, Korf I, Meyers BC, Michelmore RW (2017). Genome assembly with in vitro proximity ligation data and whole-genome triplication in lettuce. Nature Communications.

[bib104] Rhee SY, Beavis W, Berardini TZ, Chen G, Dixon D, Doyle A, Garcia-Hernandez M, Huala E, Lander G, Montoya M, Miller N, Mueller LA, Mundodi S, Reiser L, Tacklind J, Weems DC, Wu Y, Xu I, Yoo D, Yoon J, Zhang P (2003). The Arabidopsis Information Resource (TAIR): a model organism database providing a centralized, curated gateway to Arabidopsis biology, research materials and community. Nucleic Acids Research.

[bib105] Ríos JL, Francini F, Schinella GR (2015). Natural products for the treatment of type 2 diabetes mellitus. Planta Medica.

[bib106] Roach MJ, Schmidt SA, Borneman AR (2018). Purge Haplotigs: allelic contig reassignment for third-gen diploid genome assemblies. BMC Bioinformatics.

[bib107] Rogers SO, Bendich AJ (1987). Ribosomal RNA genes in plants: variability in copy number and in the intergenic spacer. Plant Molecular Biology.

[bib108] Rosen J, Gray AS (2024). Software Heritage.

[bib109] Ross JR, Nam KH, D’Auria JC, Pichersky E (1999). S-Adenosyl-L-methionine:salicylic acid carboxyl methyltransferase, an enzyme involved in floral scent production and plant defense, represents a new class of plant methyltransferases. Archives of Biochemistry and Biophysics.

[bib110] Sackton TB, Clark N (2019). Convergent evolution in the genomics era: new insights and directions. Philosophical Transactions of the Royal Society B.

[bib111] Sánchez Boado L, Fretes RM, Brumovsky LA (2015). Bioavailability and antioxidant effect of the Ilex Paraguariensis polyphenols. Nutrition & Food Science.

[bib112] Sankoff D, Zheng C (2018). Whole genome duplication in plants: implications for evolutionary analysis. Methods in Molecular Biology.

[bib113] Santesmasses D, Mariotti M, Guigó R (2017). Computational identification of the selenocysteine tRNA (tRNASec) in genomes. PLOS Computational Biology.

[bib114] Santos ECS, Bicca MA, Blum-Silva CH, Costa APR, Dos Santos AA, Schenkel EP, Farina M, Reginatto FH, de Lima TCM (2015). Anxiolytic-like, stimulant and neuroprotective effects of Ilex paraguariensis extracts in mice. Neuroscience.

[bib115] Schmieder R, Edwards R (2011). Fast identification and removal of sequence contamination from genomic and metagenomic datasets. PLOS ONE.

[bib116] Schrodinger LLC (2015).

[bib117] Scrucca L, Fop M, Murphy TB, Raftery AE (2016). mclust 5: clustering, classification and density estimation using gaussian finite mixture models. The R Journal.

[bib118] Seo HS, Song JT, Cheong JJ, Lee YH, Lee YW, Hwang I, Lee JS, Choi YD (2001). Jasmonic acid carboxyl methyltransferase: a key enzyme for jasmonate-regulated plant responses. PNAS.

[bib119] Sheng L, Chai W, Gong X, Zhou L, Cai R, Li X, Zhao Y, Jiang H, Cheng B (2015). Identification and characterization of novel maize mirnas involved in different genetic background. International Journal of Biological Sciences.

[bib120] Shui XR, Chen ZW, Li JX (2013). MicroRNA prediction and its function in regulating drought-related genes in cowpea. Plant Science.

[bib121] Singh R, Ming R, Yu Q (2016). Comparative analysis of GC content variations in plant genomes. Tropical Plant Biology.

[bib122] Song QX, Liu YF, Hu XY, Zhang WK, Ma B, Chen SY, Zhang JS (2011). Identification of miRNAs and their target genes in developing soybean seeds by deep sequencing. BMC Plant Biology.

[bib123] Song JB, Gao S, Sun D, Li H, Shu XX, Yang ZM (2013). miR394 and LCR are involved in Arabidopsis salt and drought stress responses in an abscisic acid-dependent manner. BMC Plant Biology.

[bib124] Stevenson PC, Nicolson SW, Wright GA (2017). Plant secondary metabolites in nectar: impacts on pollinators and ecological functions. Functional Ecology.

[bib125] Sun G, Stewart CN, Xiao P, Zhang B (2012). MicroRNA expression analysis in the cellulosic biofuel crop switchgrass (Panicum virgatum) under abiotic stress. PLOS ONE.

[bib126] Sun R, Guo T, Cobb J, Wang Q, Zhang B (2015). Role of microRNAs during flower and storage root development in sweet potato. Plant Molecular Biology Reporter.

[bib127] Suzuki T, Takahashi E (1976). Caffeine biosynthesis in Camellia sinensis. Phytochemistry.

[bib128] Swain M (2012). chemicalize.org. Journal of Chemical Information and Modeling.

[bib129] Tang S, Wang Y, Li Z, Gui Y, Xiao B, Xie J, Zhu QH, Fan L (2012). Identification of wounding and topping responsive small RNAs in tobacco (Nicotiana tabacum). BMC Plant Biology.

[bib130] Tarragó J, Filip R, Mroginski L, Sansberro P (2012). Influence of the irradiance on phenols content and rooting of Ilex paraguariensis cuttings collected from adult plants. Acta Physiologiae Plantarum.

[bib131] Tatusov RL, Fedorova ND, Jackson JD, Jacobs AR, Kiryutin B, Koonin EV, Krylov DM, Mazumder R, Mekhedov SL, Nikolskaya AN, Rao BS, Smirnov S, Sverdlov AV, Vasudevan S, Wolf YI, Yin JJ, Natale DA (2003). The COG database: an updated version includes eukaryotes. BMC Bioinformatics.

[bib132] Thornton JW (2004). Resurrecting ancient genes: experimental analysis of extinct molecules. Nature Reviews. Genetics.

[bib133] Thorogood CJ, Bauer U, Hiscock SJ (2018). Convergent and divergent evolution in carnivorous pitcher plant traps. The New Phytologist.

[bib134] Trifinopoulos J, Nguyen LT, von Haeseler A, Minh BQ (2016). W-IQ-TREE: a fast online phylogenetic tool for maximum likelihood analysis. Nucleic Acids Research.

[bib135] Uefuji H, Ogita S, Yamaguchi Y, Koizumi N, Sano H (2003). Molecular cloning and functional characterization of three distinct N-methyltransferases involved in the caffeine biosynthetic pathway in coffee plants. Plant Physiology.

[bib136] Varkonyi-Gasic E, Gould N, Sandanayaka M, Sutherland P, MacDiarmid RM (2010). Characterisation of microRNAs from apple (Malus domestica ’Royal Gala’) vascular tissue and phloem sap. BMC Plant Biology.

[bib137] Vieira MA, Maraschin M, Pagliosa CM, Podestá R, de Simas KN, de Amboni RM, Amante ER (2010). Phenolic acids and methylxanthines composition and antioxidant properties of mate (Ilex paraguariensis) residue. Journal of Food Science.

[bib138] Vonrhein C, Flensburg C, Keller P, Sharff A, Smart O, Paciorek W, Womack T, Bricogne G (2011). Data processing and analysis with the autoPROC toolbox. Acta Crystallographica. Section D, Biological Crystallography.

[bib139] Wan T, Liu ZM, Li LF, Leitch AR, Leitch IJ, Lohaus R, Liu ZJ, Xin HP, Gong YB, Liu Y, Wang WC, Chen LY, Yang Y, Kelly LJ, Yang J, Huang JL, Li Z, Liu P, Zhang L, Liu HM, Wang H, Deng SH, Liu M, Li J, Ma L, Liu Y, Lei Y, Xu W, Wu LQ, Liu F, Ma Q, Yu XR, Jiang Z, Zhang GQ, Li SH, Li RQ, Zhang SZ, Wang QF, Van de Peer Y, Zhang JB, Wang XM (2018). A genome for gnetophytes and early evolution of seed plants. Nature Plants.

[bib140] Wang C, Shangguan L, Kibet KN, Wang X, Han J, Song C, Fang J (2011). Characterization of microRNAs identified in a table grapevine cultivar with validation of computationally predicted grapevine miRNAs by miR-RACE. PLOS ONE.

[bib141] Wang Y, Liu YF, Wei MY, Zhang CY, Chen JD, Yao MZ, Chen L, Jin JQ (2023). Deeply functional identification of *TCS1* alleles provides efficient technical paths for low-caffeine breeding of tea plants. Horticulture Research.

[bib142] Xie Z, Allen E, Fahlgren N, Calamar A, Givan SA, Carrington JC (2005). Expression of arabidopsis MIRNA genes. Plant Physiology.

[bib143] Xie F, Frazier TP, Zhang B (2011). Identification, characterization and expression analysis of MicroRNAs and their targets in the potato (Solanum tuberosum). Gene.

[bib144] Xie F, Stewart CN, Taki FA, He Q, Liu H, Zhang B (2014). High-throughput deep sequencing shows that microRNAs play important roles in switchgrass responses to drought and salinity stress. Plant Biotechnology Journal.

[bib145] Xu KW, Wei XF, Lin CX, Zhang M, Zhang Q, Zhou P, Fang YM, Xue JY, Duan YF (2022). The chromosome-level holly (*Ilex latifolia*) genome reveals key enzymes in triterpenoid saponin biosynthesis and fruit color change. Frontiers in Plant Science.

[bib146] Yang Z (1995). A space-time process model for the evolution of DNA sequences. Genetics.

[bib147] Yang X, Hu R, Yin H, Jenkins J, Shu S, Tang H, Liu D, Weighill DA, Cheol Yim W, Ha J, Heyduk K, Goodstein DM, Guo HB, Moseley RC, Fitzek E, Jawdy S, Zhang Z, Xie M, Hartwell J, Grimwood J, Abraham PE, Mewalal R, Beltrán JD, Boxall SF, Dever LV, Palla KJ, Albion R, Garcia T, Mayer JA, Don Lim S, Man Wai C, Peluso P, Van Buren R, De Paoli HC, Borland AM, Guo H, Chen JG, Muchero W, Yin Y, Jacobson DA, Tschaplinski TJ, Hettich RL, Ming R, Winter K, Leebens-Mack JH, Smith JAC, Cushman JC, Schmutz J, Tuskan GA (2017). The Kalanchoë genome provides insights into convergent evolution and building blocks of crassulacean acid metabolism. Nature Communications.

[bib148] Yang L, Su D, Chang X, Foster CSP, Sun L, Huang CH, Zhou X, Zeng L, Ma H, Zhong B (2020). Phylogenomic insights into deep phylogeny of angiosperms based on broad nuclear gene sampling. Plant Communications.

[bib149] Yao X, Song Y, Yang JB, Tan YH, Corlett RT (2021). Phylogeny and biogeography of the hollies ( *Ilex* L., Aquifoliaceae). Journal of Systematics and Evolution.

[bib150] Yao X, Lu Z, Song Y, Hu X, Corlett RT (2022). A chromosome-scale genome assembly for the holly (Ilex polyneura) provides insights into genomic adaptations to elevation in Southwest China. Horticulture Research.

[bib151] Yin Y, Katahira R, Ashihara H (2015). Metabolism of purine alkaloids and xanthine in leaves of maté (Ilex paraguariensis). Natural Product Communications.

[bib152] Yoneyama N, Morimoto H, Ye CX, Ashihara H, Mizuno K, Kato M (2006). Substrate specificity of N-methyltransferase involved in purine alkaloids synthesis is dependent upon one amino acid residue of the enzyme. Molecular Genetics and Genomics.

[bib153] Zan T, He YT, Zhang M, Yonezawa T, Ma H, Zhao QM, Kuo WY, Zhang WJ, Huang CH (2023). Phylogenomic analyses of Camellia support reticulate evolution among major clades. Molecular Phylogenetics and Evolution.

[bib154] Zhang L, Zheng Y, Jagadeeswaran G, Li Y, Gowdu K, Sunkar R (2011). Identification and temporal expression analysis of conserved and novel microRNAs in Sorghum. Genomics.

[bib155] Zhang Y, Zhu X, Chen X, Song C, Zou Z, Wang Y, Wang M, Fang W, Li X (2014). Identification and characterization of cold-responsive microRNAs in tea plant (Camellia sinensis) and their targets using high-throughput sequencing and degradome analysis. BMC Plant Biology.

[bib156] Zhang YH, Li YF, Wang Y, Tan L, Cao ZQ, Xie C, Xie G, Gong HB, Sun WY, Ouyang SH, Duan WJ, Lu X, Ding K, Kurihara H, Hu D, Zhang ZM, Abe I, He RR (2020a). Identification and characterization of N9-methyltransferase involved in converting caffeine into non-stimulatory theacrine in tea. Nature Communications.

[bib157] Zhang C, Zhang T, Luebert F, Xiang Y, Huang CH, Hu Y, Rees M, Frohlich MW, Qi J, Weigend M, Ma H (2020b). Asterid phylogenomics/phylotranscriptomics uncover morphological evolutionary histories and support phylogenetic placement for numerous whole-genome duplications. Molecular Biology and Evolution.

[bib158] Zhao N, Ferrer JL, Ross J, Guan J, Yang Y, Pichersky E, Noel JP, Chen F (2008). Structural, biochemical, and phylogenetic analyses suggest that indole-3-acetic acid methyltransferase is an evolutionarily ancient member of the SABATH family. Plant Physiology.

[bib159] Zhao CZ, Xia H, Frazier TP, Yao YY, Bi YP, Li AQ, Li MJ, Li CS, Zhang BH, Wang XJ (2010). Deep sequencing identifies novel and conserved microRNAs in peanuts (Arachis hypogaea L.). BMC Plant Biology.

[bib160] Zhao C, Xia H, Cao T, Yang Y, Zhao S, Hou L, Zhang Y, Li C, Zhang X, Wang X (2015). Small RNA and degradome deep sequencing reveals peanut MicroRNA roles in response to pathogen infection. Plant Molecular Biology Reporter.

[bib161] Zhu Q, Luo Y (2013). Identification of miRNAs and their targets in tea (Camellia sinensis). Journal of Zhejiang University. Science. B.

[bib162] Zubieta C, Ross JR, Koscheski P, Yang Y, Pichersky E, Noel JP (2003). Structural basis for substrate recognition in the salicylic acid carboxyl methyltransferase family. The Plant Cell.

