# Supplementary material for: Yerba mate (Ilex paraguariensis) genome provides new insights into convergent evolution of caffeine biosynthesis
Source: eLife. 2025 Jan 8;14:e104759. doi: 10.7554/eLife.104759 (PMC11709435; doi:10.7554/eLife.104759)
Supplement: Figure 3—source data 1. [file elife-104759-fig3-data1.docx]

| **Figure 3–Source Data 1**. Accession numbers of SABATH sequences used for phylogenetic analysis in Figure 3. | | |
| --- | --- | --- |
| **Accession Number** | **Gene** | **Species** |
| ABV91100.1 | CCMT1 | *Ocimum basilicum* |
| NP_001406848.1 | IAMT1 | *Oryza sativa subsp. japonica* |
| NP_200336.1 | IAMT1 | *Arabidopsis thaliana* |
| KAH9652080.1 | IAMT1 | *Citrus sinensis* |
| XP_002298843.1 | IAMT1 | *Populus trichocarpa* |
| NP_194372.2 | GAMT1 | *Arabidopsis thaliana* |
| NP_200441.2 | GAMT2 | *Arabidopsis thaliana* |
| NP_190072.1 | FAMT | *Arabidopsis thaliana* |
| gnl\|onekp\|DAAD_scaffold_2041891 | FAMT | *Ardisia revoluta* |
| B2KPR3.1 | LAMT | *Catharanthus roseus* |
| XP_015624979.1 | BSMT | *Oryza sativa subsp. japonica* |
| D9J0Z7.1 | AAMT1 | *Zea mays* |
| NP_187755.2 | BSMT1 | *Arabidopsis thaliana* |
| AAP57211.1 | BSMT1 | *Arabidopsis lyrata subsp. lyrata* |
| gnl\|onekp\|VKGP_scaffold_2120585 | CS | *Geranium carolinianum* |
| gnl\|onekp\|YGCX_scaffold_2142952 | CS | *Geranium maculatum* |
| EC774687 | CS0 | *Paullinia cupana var. sorbilis* |
| EC778019 | CS2 | *Paullinia cupana var. sorbilis* |
| EC766748 | CS1 | *Paullinia cupana var. sorbilis* |
| DAA64605.1 | CS | *Paullinia cupana var. sorbilis* |
| gnl\|onekp\|VFFP_scaffold_2043613 | CS | *Acer negundo* |
| gnl\|onekp\|WAXR_scaffold_2040211 | CS | *Litchi chinensis* |
| KDO69071.1 | CS | *Citrus sinensis* |
| GBCV01000539.1 | CS | *Mangifera indica* |
| gnl\|onekp\|YUOM_scaffold_2035237 | CS | *Rhus radicans* |
| gnl\|onekp\|BCAA_scaffold_2069913 | CS | *Kirkia wilmsii* |
| gnl\|onekp\|FCCA_scaffold_2008319 | CS | *Boswellia sacra* |
| KAJ4720014.1 | CS | *Melia azedarach* |
| gnl\|onekp\|WMUK_scaffold_2093724 | CS | *Schizolaena* sp. |
| gnl\|onekp\|ATFX_scaffold_2034890 | CS | *Muntingia calabura* |
| BAE79730.1 | BTS1 | *Theobroma cacao* |
| A0A061FKL9.1 | CS1 | *Theobroma cacao* |
| A0A061FKM4.1 | CS2 | *Theobroma cacao* |
| Q68CM3.1 | CS2 | *Camellia sinensis* |
| Q9FZN8.1 | CS1 | *Camellia sinensis* |
| gnl\|onekp\|ADHK_scaffold_2052625 | CS | *Galax urceolata* |
| gnl\|onekp\|PPPZ_scaffold_2019622 | CS | *Rhododendron scopulorum* |
| gnl\|onekp\|ODDO_scaffold_2102454 | CS | *Ardisia humilis* |
| gnl\|onekp\|WMUK_scaffold_2020889 | CS | *Schizolaena* sp. |
| Q9SPV4.1 | SAMT | *Clarkia breweri* |
| AAN40745.1 | SAMT | *Antirrhinum majus* |
| CAI05934.1 | SAMT | *Hoya carnosa* |
| ACZ55216.1 | SAMT | *Nicotiana suaveolens* |
| NP_001289539.1 | SAMT | *Nicotiana sylvestris* |
| ACZ55219.1 | SAMT | *Nicotiana alata* |
| EF472972.1 | SAMT | *Datura wrightii* |
| BAB39396.1 | SAMT | *Atropa belladonna* |
| KAH9711297.1 | SAMT | *Citrus sinensis* |
| ACZ55224.1 | NAMT | *Nicotiana gossei* |
| AJ628349.1 | BSMT1 | *Nicotiana suaveolens* |
| NA | NAMT | *Nicotiana suaveolens* |
| ACZ55223.1 | BSMT2 | *Nicotiana sylvestris* |
| ACZ55220.1 | BSMT2 | *Nicotiana alata* |
| ACZ55217.1 | BSMT2 | *Nicotiana suaveolens* |
| AAG23343.1 | JMT | *Arabidopsis thaliana* |
| XP_002307671.1 | JMT | *Populus trichocarpa* |
| XP_004291853.1 | JMT2 | *Fragaria vesca subsp. vesca* |
| XP_004291852.1 | JMT1 | *Fragaria vesca subsp. vesca* |
| Q9FYZ9.1 | BAMT | *Antirrhinum majus* |
| KDO50937.1 | XMT1 | *Citrus sinensis* |
| KDO40396.1 | XMT2 | *Citrus sinensis* |
| XM_006469387.4 | XMTB | *Citrus sinensis* |
| XM_024190084.1 | XMTA | *Citrus sinensis* |
| AFV60438.1 | DXMT1 | *Coffea arabica* |
| XP_027086771.1 | XMT1 | *Coffea arabica* |
| NP_001392358.1 | MXMT1 | *Coffea arabica* |
| Q84PP7.1 | MXMT2 | *Coffea arabica* |
